# *SMAD4* mutations do not preclude epithelial–mesenchymal transition in colorectal cancer

**DOI:** 10.1038/s41388-021-02128-2

**Published:** 2021-12-03

**Authors:** Patrick Frey, Antoine Devisme, Katja Rose, Monika Schrempp, Vivien Freihen, Geoffroy Andrieux, Melanie Boerries, Andreas Hecht

**Affiliations:** 1 Institute of Molecular Medicine and Cell Research, Faculty of Medicine, University of Freiburg, Freiburg, Germany; 2 Spemann Graduate School of Biology and Medicine (SGBM), University of Freiburg, Freiburg, Germany; 3 Faculty of Biology, University of Freiburg, Freiburg, Germany; 4 Institute of Medical Bioinformatics and Systems Medicine, Medical Center – University of Freiburg, Faculty of Medicine, University of Freiburg, Freiburg, Germany; 5 German Cancer Consortium (DKTK), partner site Freiburg, Germany, and German Cancer Research Center (DKFZ), Heidelberg, Germany; 6 BIOSS Centre for Biological Signalling Studies, University of Freiburg, Freiburg, Germany

**Keywords:** Cancer genetics, Cell signalling, Mechanisms of disease

## Abstract

Transforming growth factor beta (TGFβ) superfamily signaling is a prime inducer of epithelial-mesenchymal transitions (EMT) that foster cancer cell invasion and metastasis, a major cause of cancer-related deaths. Yet, TGFβ signaling is frequently inactivated in human tumor entities including colorectal cancer (CRC) and pancreatic adenocarcinoma (PAAD) with a high proportion of mutations incapacitating *SMAD4*, which codes for a transcription factor (TF) central to canonical TGFβ and bone morphogenetic protein (BMP) signaling. Beyond its role in initiating EMT, SMAD4 was reported to crucially contribute to subsequent gene regulatory events during EMT execution. It is therefore widely assumed that *SMAD4*-mutant (*SMAD4*^mut^) cancer cells are unable to undergo EMT. Here, we scrutinized this notion and probed for potential SMAD4-independent EMT execution using *SMAD4*^mut^ CRC cell lines. We show that *SMAD4*^mut^ cells exhibit morphological changes, become invasive, and regulate EMT marker genes upon induction of the EMT-TF SNAIL1. Furthermore, SNAIL1-induced EMT in *SMAD4*^mut^ cells was found to be entirely independent of TGFβ/BMP receptor activity. Global assessment of the SNAIL1-dependent transcriptome confirmed the manifestation of an EMT gene regulatory program in *SMAD4*^mut^ cells highly related to established EMT signatures. Finally, analyses of human tumor transcriptomes showed that *SMAD4* mutations are not underrepresented in mesenchymal tumor samples and that expression patterns of EMT-associated genes are similar in *SMAD4*^mut^ and *SMAD4* wild-type (*SMAD4*^wt^) cases. Altogether, our findings suggest that alternative TFs take over the gene regulatory functions of SMAD4 downstream of EMT-TFs, arguing for considerable plasticity of gene regulatory networks operating in EMT execution. Further, they establish that EMT is not categorically precluded in *SMAD4*^mut^ tumors, which is relevant for their diagnostic and therapeutic evaluation.

## Introduction

The TGFβ superfamily comprises the TGFβ and BMP signaling pathways, whose activities have profound effects in developmental processes, adult tissue homeostasis, injury and wound healing, but also in disease [1, 2]. TGFβ/BMP signaling processes are initiated upon ligand-induced complex formation by two pairs of type I and type II transmembrane receptor proteins with intrinsic serine/threonine kinase activity [1, 2]. Following tetramerization and phosphorylation by type II subunits, type I receptors recognize and phosphorylate receptor-regulated or R-SMAD proteins. These are SMAD2 and SMAD3 (SMAD2/3) in case of TGFβ receptors, and SMAD1, SMAD5, and SMAD8 (SMAD1/5/8) for BMP receptors. Phosphorylated R-SMADs form trimeric TF complexes with a common binding partner, SMAD4, accumulate in the nucleus, occupy specific regulatory DNA sequence elements, and engage in control of target gene transcription. Through its interaction with both classes of R-SMADs, SMAD4 therefore occupies a central role in TGFβ and BMP receptor-mediated signaling and is connected to most but not all TGFβ superfamily responses. Also, aside from canonical, SMAD-dependent processes, TGFβ and BMP receptors can trigger SMAD-independent cellular responses by signaling via mitogen-activated protein kinases and other signal transducers [1–3].

In human carcinogenesis, both gain-of-function and loss-of-function of TGFβ pathway activity are observed, affecting cancer cells and stromal cells alike [1, 4]. Particularly high frequencies of mutations in the TGFβ pathway are found in CRC and PAAD [5, 6]. In these entities, *SMAD4* represents the most commonly inactivated TGFβ pathway component. *SMAD4* mutation rates are around 20% in CRC [5] and 30% in PAAD [6] and are associated with poor clinical prognosis for patients [7, 8]. Mechanistically, the high frequency of disabling TGFβ pathway mutations in cancer cells can be explained by the growth-inhibitory and pro-apoptotic functions of this signaling pathway [1, 4]. Yet, besides its tumor-suppressing effects, TGFβ signaling can also be tumor-promoting, acting as a widespread and potent inducer of EMT which is linked to cancer cell invasion and metastasis [1, 9].

EMT entails the acquisition of migratory and invasive properties at the expense of epithelial cell-cell adhesion and apical-basal polarity [9] and is controlled by a group of master regulators, the so-called EMT-TFs [9, 10]. The expression of EMT-TFs that comprise the SNAIL, ZEB and TWIST protein families, can be induced by several signaling pathways and conditions [9–12]. However, TGFβ signaling arguably is the best characterized and most widely implicated inducer of EMT with EMT-TFs being direct and indirect targets of TGFβ-activated SMAD complexes [9, 10, 13–16]. Moreover, beyond their classical function in triggering EMT, TGFβ superfamily signaling and SMAD4 play additional roles during the unfolding of EMT processes in cooperation with and downstream of EMT-TFs [15, 17–19]. For example, SMAD4 contributes to the downregulation of the epithelial marker gene *CDH1* (E-CADHERIN) by forming a transcriptional repressor complex with the EMT-TF SNAIL1 [18]. Furthermore, we recently reported that, following SNAIL1 expression, BMP pathway activity and SMAD4 were indispensable for the implementation of EMT in a *SMAD4*^wt^ background [19]. TGFβ superfamily signaling and in particular SMAD4 are therefore typically considered to be pivotal for both the induction as well as the execution of EMT. Accordingly, frequently occurring *SMAD4* mutations might prevent EMT for two reasons. First, because SMAD4 is not available for TGFβ-induced upregulation of core EMT-TFs, and second because SMAD4-deficiency should impair EMT execution downstream of EMT-TFs.

In light of the dual function of TGFβ signaling and SMAD4 in EMT processes, one could assume that mutations in TGFβ pathway components categorically preclude the occurrence of EMT in human cancer cells. Recently, however, several studies suggested that the mechanistic routes to EMT can be subject to considerable plasticity [14, 20, 21]. Therefore, it is conceivable that in *SMAD4*^mut^ tumors alternative signaling pathways and TFs might be engaged in the induction of EMT-TFs and the execution of EMT. In the present study, we aimed to explicitly test the hypothesis of SMAD4-independent EMT execution. We report that two epithelial *SMAD4*^mut^ CRC cell lines acquire mesenchymal characteristics and regulate EMT marker genes in response to SNAIL1 induction. Additionally, we demonstrate that the acquired EMT phenotype is independent of TGFβ and BMP receptor activity in general. By analyzing the global transcriptomic changes during SNAIL1-induced EMT in *SMAD4*^mut^ cells, we establish their similarity to multiple published EMT core gene signatures [20, 22–24] and to the gene expression profile of consensus molecular subtype 4 (CMS4) of human CRC, which is characterized by a mesenchymal phenotype and highly unfavorable prognosis [25]. Moreover, we show that *SMAD4* mutations are not underrepresented in the most mesenchymal CRC and PAAD tumors and do not abrogate the elevated expression of EMT-TFs in EMT-associated samples. Collectively, our results indicate that mutations in TGFβ pathway components, like *SMAD4*, do not preclude EMT in CRC. Hence, we propose that in *SMAD4*^mut^ backgrounds, alternative TFs take over the gene regulatory functions of SMAD4 during EMT.

## Results

### *SMAD4*^mut^ CRC cell lines undergo EMT-associated morphological changes in the presence of SNAIL1

**Fig. 1:**
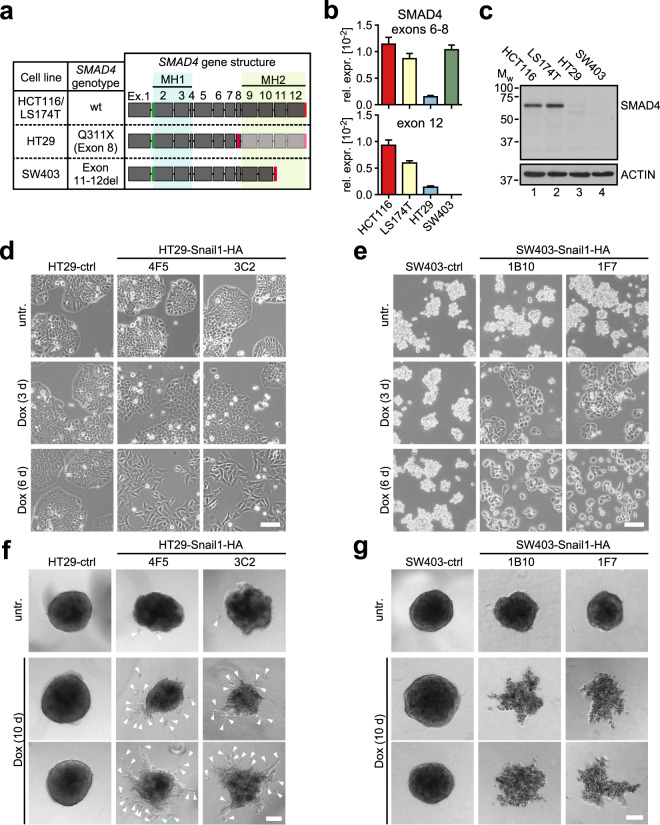
HT29 and SW403 CRC cell lines are SMAD4-negative but show morphological evidence of EMT upon Snail1-HA overexpression. **a** *SMAD4* mutation status in four CRC cell lines and schematic depictions of respective gene structures and their coding capacities. Exon numbers as well as locations of the two major functional domains MH1 and MH2 are given on top. Positions of start codons are marked in green, stop codons are indicated in red. Introns are not drawn to scale. The precise location of the stop codon in SW403 cells is not known. **b** Analyses of *SMAD4* mRNA expression by qRT-PCR in the four CRC cell lines. Primers were located in exons 6 and 8, and in exon 12. Relative gene expression (rel. expr.) was calculated by normalizing to the expression of *GAPDH*. Plotted is the mean + SEM; *n* = 3. **c** Analyses of SMAD4 protein expression in the four CRC cell lines by immunoblotting. Positions of molecular weight (Mw) standards in kDa are indicated on the left. ACTIN was detected as a loading control. **d** Representative phase contrast images of HT29-ctrl cells and HT29-Snail1-HA clones 4F5 and 3C2. Cells were left untreated (untr.) or received Dox for the indicated time spans. Scale bar: 100 µm. **e** Representative phase contrast images of SW403-ctrl cells and SW403-Snail1-HA clones 1B10 and 1F7. Cells were left untreated or received Dox for the indicated time spans. Scale bar: 100 µm. **f** Representative spheroids formed by HT29-ctrl cells and HT29-Snail1-HA clones 4F5 and 3C2 in a collagen I matrix after treatment with Dox as indicated. Cells that are separated from the spheroid bodies are highlighted by white arrowheads and were counted for quantification of cell invasiveness (see Fig. S2e). Scale bar: 100 µm. **g** Representative spheroids formed by SW403-ctrl cells and SW403-Snail1-HA clones 1B10 and 1F7 in a collagen I matrix after treatment with Dox as indicated. Scale bar: 100 µm.

As model systems to investigate the impact of *SMAD4*^mut^ conditions on EMT processes, we employed two epithelial CRC cell lines which carry homozygous mutations in the *SMAD4* gene described to completely abrogate SMAD4 protein expression [26]. HT29 cells have a nonsense mutation in *SMAD4* exon 8 (Fig. 1a) disrupting the reading frame upstream of the MH2 domain, which is required for SMAD complex formation. SW403 cells have a deletion of *SMAD4* exons 11 and 12, which also affects the coding region for the MH2 domain. First, we examined *SMAD4* transcript levels and SMAD4 protein expression in the *SMAD4*^mut^ cell lines. Compared to HCT116 and LS174T CRC cells which were used as *SMAD4*^wt^ controls, we measured lower *SMAD4* RNA levels in HT29 cells. In SW403 cells, we detected wild-type levels of *SMAD4* transcripts with a primer pair spanning exons 6 to 8, while no transcripts could be found with primers located in exon 12. These findings are consistent with the *SMAD4* genomic structure in SW403 cells (Fig. 1a, b). Importantly, no SMAD4 protein expression could be observed in HT29 and SW403 cells in Western blot experiments using a SMAD4 antibody that recognizes an epitope encoded by *SMAD4* exon 5 and thus would have allowed detection of shorter protein forms which might have potentially been generated from the mutant alleles [26] (Fig. 1c).

The absence of SMAD4 should abrogate the ability of TGFβ superfamily signaling to function as inducer of EMT upstream of EMT-TFs. To verify this, we monitored BMP and TGFβ receptor activity in HT29 and SW403 cells and performed growth factor stimulations. We found that untreated HT29 and SW403 cells exhibited considerable BMP receptor activity indicated by the presence of phospho-SMAD1/5/8 (Fig. S1). This activity could not be further increased by administration of BMP4. Apparently, though, active BMP signaling did not interfere with the epithelial state of HT29 and SW403 cells (Fig. S1). In contrast, using SMAD2/3 phosphorylation as read-out, we could detect TGFβ receptor activity in HT29 and SW403 cells only upon TGFβ1 administration but not in untreated cells (Fig. S1). Despite TGFβ pathway activation, neither cell line exhibited changes in cell shape and gene expression indicative of EMT, bolstering the idea that TGFβ signaling has no EMT-inducing capacity in *SMAD4*^mut^ cells.

To test whether SMAD4 deficiency also results in defective EMT execution downstream of EMT-TFs, we transduced HT29 and SW403 cells with a construct allowing for doxycycline (Dox)-inducible expression of murine HA-tagged Snail1 (Snail1-HA). However, when we analyzed the morphology of the cells after Dox treatment, HT29-Snail1-HA and SW403-Snail1-HA polyclonal cell populations showed markedly heterogeneous changes in cell shape with only a fraction of the cells adopting a spindle-like appearance indicative of EMT (Figs. S2a, S3a). This heterogeneity could be caused by failure of the Snail1-HA expression construct in some of the transduced cells, but also by clonal differences in susceptibility to Snail1-HA-induced EMT. To investigate this, we generated HT29-Snail1-HA and SW403-Snail1-HA single-cell clones and tested their response to Dox treatment (Figs. S2b, S3b). For each cellular background we identified cell clones which upon Dox administration homogeneously shifted to a mesenchymal phenotype, but also some which did not respond. Notably, non-responding clones did not induce Snail1-HA after Dox treatment or showed much lower Snail1-HA expression than the responders (Figs. S2c, S3c). Furthermore, non-responding cell clones also did not show deregulation of epithelial and mesenchymal marker genes (Figs. S2c, S3c). In contrast, responding clones displayed strong Snail1-HA induction and the expected regulation of EMT marker genes at amplitudes exceeding those observed in the parental polyclonal cell populations. Importantly, for the HT29 cells, we excluded *SMAD4*^mut^ reversion in clones responding to Snail1-HA expression (Fig. S2d). These clear correlations between Snail1-HA expression levels and ensuing changes in gene expression and cell shape argue for cell clone-specific failure of the overexpression construct and indicate that *SMAD4*^mut^ HT29 and SW403 cells retain the ability to undergo EMT in the presence of an EMT-TF. To further study the effects of Snail1-HA overexpression, we therefore chose two HT29-Snail1-HA clones (4F5, 3C2) and two SW403-Snail1-HA clones (1B10, 1F7).

Time-resolved microscopic examination in 2D culture conditions revealed that both HT29-Snail1-HA clones showed morphological changes indicative of EMT after 6 d of Dox treatment. Cell clusters first flattened and then dispersed into individual cells taking on a spindle-like cell shape (Fig. 1d). This morphological transition was also seen in the SW403-Snail1-HA clones (Fig. 1e). Using transwell migration assays we observed increased motility of Snail1-HA-expressing HT29 cells but not of SW403 cells (Fig. S4). Possibly, this difference is due to absent expression of FIBRONECTIN or other factors involved in cell-matrix adhesion in SW403 cells (see below). Nonetheless, Dox-induced HT29-Snail1-HA and SW403-Snail1-HA clones exhibited signs of invasiveness when embedded as multicellular spheroids in a 3D collagen I matrix. Upon expression of Snail1-HA, HT29 spheroids exhibited formation of cell strands radiating from the spheroid bodies and detachment of single cells which invaded the surrounding matrix (Figs. 1f and S2e). Compared to untreated controls, spheroids derived from Dox-induced SW403-Snail1-HA cells displayed irregular shapes with more clearly distinguishable cell borders and evidence of invasive cell strand formation (Fig. 1g). Importantly, despite their disintegrating appearance, SW403-Snail1-HA-derived spheroids were viable as evidenced by MTT staining (Fig. S3d). In conclusion, HT29 and SW403 CRC cell lines showed morphological and behavioral signs of EMT in 2D and 3D environments after overexpression of Snail1-HA even though they both lack SMAD4 expression due to *SMAD4* mutations.

### EMT marker genes are deregulated in HT29 and SW403 cells after Snail1-HA induction

**Fig. 2:**
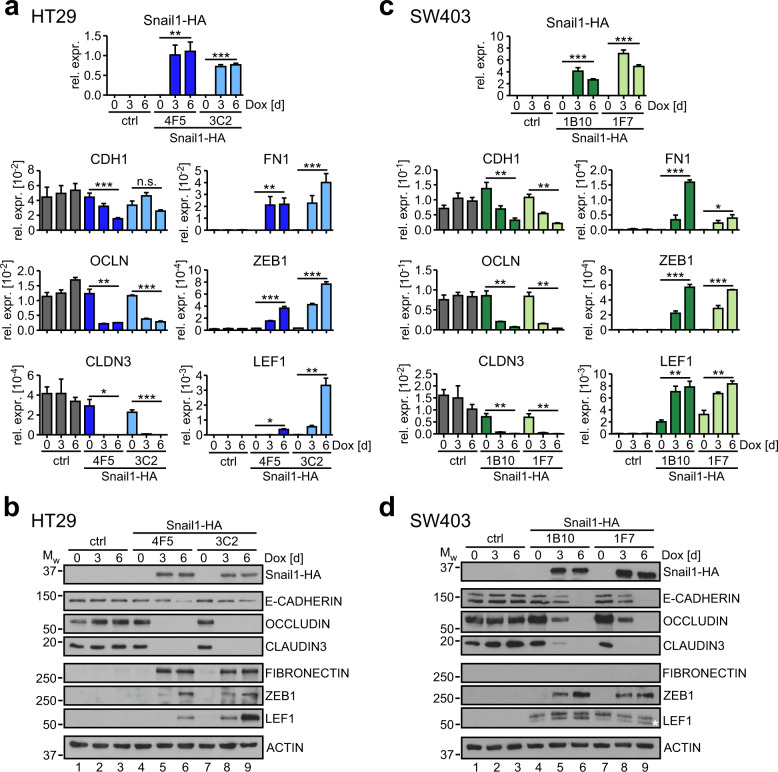
The expression of EMT marker genes is changed in HT29 and SW403 CRC cells after Snail1-HA induction. **a** Analyses of mRNA expression by qRT-PCR in HT29-ctrl cells and HT29-Snail1-HA clones 4F5 and 3C2 treated with Dox as indicated. Relative gene expression (rel. expr.) was calculated by normalizing to the expression of *GAPDH*. Plotted is the mean + SEM; *n* ≥ 3. Two-tailed student’s *t*-test; *p-value < 0.05, **p-value < 0.01, ***p-value < 0.001. **b** Analyses of protein expression by immunoblotting in HT29-ctrl cells and HT29-Snail1-HA clones 4F5 and 3C2 treated with Dox as indicated. Positions of molecular weight (Mw) standards in kDa are indicated on the left. One representative loading control of ACTIN is shown for reasons of simplicity. All loading controls corresponding to the depicted protein detections are given in Fig. S9a. A quantification of E-CADHERIN protein levels can be found in Fig. S5c. **c** Analyses of mRNA expression by qRT-PCR in SW403-ctrl cells and SW403-Snail1-HA clones 1B10 and 1F7 that were treated with Dox as indicated. Relative gene expression (rel. expr.) was calculated by normalizing to the expression of *GAPDH*. Plotted is the mean + SEM; *n* = 3. Two-tailed student’s *t*-test; *p-value < 0.05, **p-value < 0.01, ***p-value < 0.001. **d** Analyses of protein expression by immunoblotting in SW403-ctrl cells and SW403-Snail1-HA clones 1B10 and 1F7 that were treated with Dox as indicated. Positions of molecular weight (Mw) standards in kDa are indicated on the left. One representative loading control of ACTIN is shown for reasons of simplicity. All loading controls corresponding to the depicted protein detections are given in Fig. S9b. FIBRONECTIN could not be detected. Asterisk: Snail1-HA-responsive lower molecular weight LEF1 isoform. A quantification of E-CADHERIN protein levels can be found in Fig. S5c.

Next, we investigated whether the morphological changes observed in the presence of Snail1-HA were paralleled by changes in gene expression typical for EMT processes. For this, we analyzed the expression of the epithelial marker genes *CDH1* (E-CADHERIN), *OCLN* (OCCLUDIN), and *CLDN3* (CLAUDIN-3). Likewise, we monitored expression of four genes associated with the mesenchymal state, *FN1* (FIBRONECTIN), *ZEB1*, *LEF1*, and *FSCN1* (FASCIN1) [27, 28]. Consistent with EMT, epithelial markers were downregulated and mesenchymal markers were upregulated in the HT29-Snail1-HA and SW403-Snail1-HA clones at RNA and protein levels in response to Dox treatment (Figs. 2 and S5a–c). An exception was *FN1* whose upregulation in SW403-Snail1-HA cells was only detectable at RNA level. In case of LEF1 and FASCIN1, both SW403-Snail1-HA clones and SW403-Snail1-HA clone 1F7, respectively, showed elevated expression already in the uninduced state, which, however, could still be boosted by Snail1-HA (Figs. 2c, d and S5a, b). For LEF1, specifically expression of a lower molecular weight variant seemed to be affected (Fig. 2d). In addition to measuring bulk RNA and protein expression changes we performed immunofluorescence analyses for selected epithelial and mesenchymal markers. These confirmed decreased overall levels and revealed diminished membrane staining of E-CADHERIN and CLAUDIN3 in Dox-treated cells, while cytoplasmic signals for the actin bundling protein FASCIN1 increased (Fig. S5d, e). Altogether, targeted analyses of gene expression showed that EMT marker genes are deregulated in response to Snail1-HA induction in HT29 and SW403 cells despite their *SMAD4*^mut^ status.

### The EMT phenotype of HT29-Snail1-HA cells is independent of BMP and TGFβ receptor activity

**Fig. 3:**
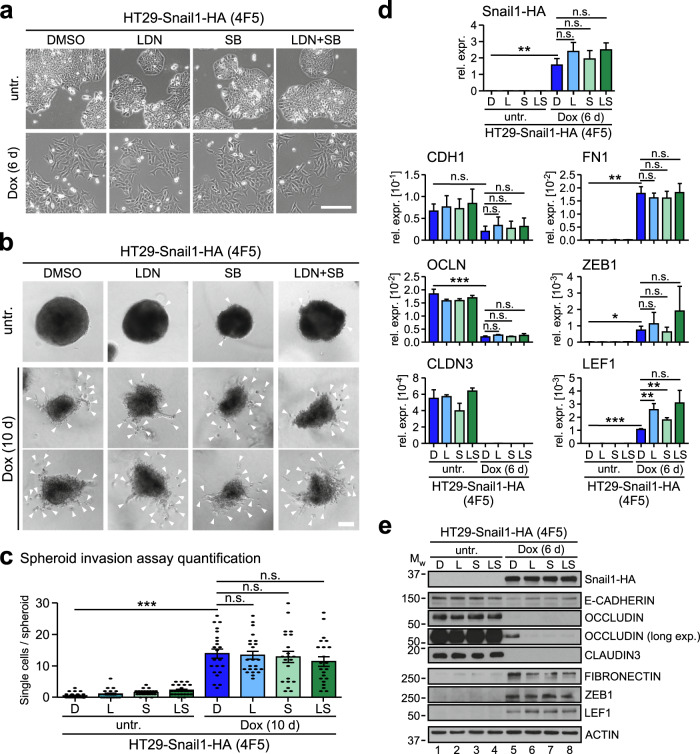
EMT in HT29-Snail1-HA cells occurs independently of BMP and TGFβ receptor activities. **a** Representative phase contrast images of the HT29-Snail1-HA clone 4F5 cells treated with Dox and DMSO, LDN193189 (LDN), SB431542 (SB), or a combination of LDN and SB as indicated. Scale bar: 200 µm. **b** Representative spheroids formed by HT29-Snail1-HA clone 4F5 cells in a collagen I matrix. Cells were treated with Dox and DMSO, LDN, SB, or a combination of LDN and SB as indicated. Cells that are separated from the spheroid bodies are highlighted by white arrowheads and were counted for quantification of cell invasiveness shown in **c**. Scale bar: 100 µm. **c** Quantification of spheroid invasion assays with HT29-Snail1-HA clone 4F5 cells by manual counting of single cells that were separated from the spheroid body. Representative spheroid images are shown in **b**. A total number of ≥21 spheroids compiled from three biological replicates were quantified for each condition. Two-tailed Mann–Whitney U test; n.s.: not significant, ***p-value < 0.001. **d** Analyses of mRNA expression by qRT-PCR in HT29-Snail1-HA clone 4F5 cells treated with Dox and DMSO (D), LDN (L), SB (S), or a combination of LDN and SB (LS) as indicated. Relative gene expression (rel. expr.) was calculated by normalizing to the expression of *GAPDH*. Plotted is the mean + SEM; *n* = 3. Two-tailed student’s *t*-test; *p-value < 0.05, **p-value < 0.01, ***p-value < 0.001. **e** Analyses of *p*rotein expression by immunoblotting in HT29-Snail1-HA clone 4F5 cells treated with Dox and DMSO (D), LDN (L), SB (S), or a combination of LDN and SB (LS) as indicated. Positions of molecular weight (Mw) standards in kDa are indicated on the left. One representative loading control of ACTIN is shown for reasons of simplicity. All loading controls corresponding to the protein detections depicted are given in Fig. S9c. In case of the OCCLUDIN detection, a longer exposure (long exp.) is additionally shown.

Expression of SNAIL1 can lead to activation of TGFβ and BMP signaling which then may promote EMT execution [17, 19]. Further, TGFβ and BMP receptors signal intracellularly also independently of SMAD4 and SMAD-containing protein complexes via non-canonical routes [3] and thereby can affect EMT [29]. Thus, the demonstration that SMAD4 is dispensable for EMT-associated changes in morphology and gene expression after Snail1-HA overexpression, does not altogether rule out that BMP and TGFβ receptor downstream signaling contributes to Snail1-HA-induced phenotypic alterations. Consequently, we investigated whether Snail1-HA expression affected BMP and TGFβ pathway activities using phosphorylation of SMAD proteins as read-outs. However, also upon Dox-treatment no TGFβ receptor activity was detectable in HT29 cells (Fig. S6a). In contrast, similar to our previous observations in SMAD4-proficient cell line models [19], Snail1-HA overexpression further increased pre-existing BMP receptor activity (Fig. S6a). To test whether basal or Snail1-HA-potentiated BMP receptor activity affected Snail1-HA-induced EMT of HT29 cells, we used LDN193189 (LDN), an inhibitor of the type I BMP receptor BMPR1A. For comparison, we performed experiments with SB431542 (SB) which inhibits the type I TGFβ receptor TGFBR1A. As expected, basal and Snail1-HA-induced BMP receptor activities could be fully blocked by LDN but not by SB (Fig. S6a). Despite effective receptor inhibition, HT29-ctrl cell morphology did not change upon application of LDN, neither individually nor in combination with SB (Fig. S6b). Likewise, the morphological changes and single-cell invasion elicited by Dox-induced Snail1-HA expression in 2D and 3D cultures of HT29-Snail1-HA clone 4F5 were unaffected by the BMP and TGFβ receptor inhibitors (Fig. 3a–c). Furthermore, individual and combinatorial application of LDN and SB did not impair the up- and downregulation of mesenchymal and epithelial marker genes, respectively, caused by Snail1-HA (Fig. 3d). Only at the protein level, there seemed to be minor, but opposing, effects, specifically of BMP pathway inhibition, on the amounts of E-CADHERIN, FIBRONECTIN, LEF1, and OCCLUDIN (Fig. 3e). In summary, we conclude that inhibition of TGFβ superfamily receptors does not impair the Snail1-HA-induced phenotypic changes and the regulation of EMT marker genes in HT29-Snail1-HA cells. EMT execution in these cells therefore occurs not only independently of SMAD4, but also of BMP and TGFβ pathway activities *in toto*.

### Global transcriptome analyses confirm Snail1-HA-induced EMT of *SMAD4*^mut^ HT29 cells

**Fig. 4:**
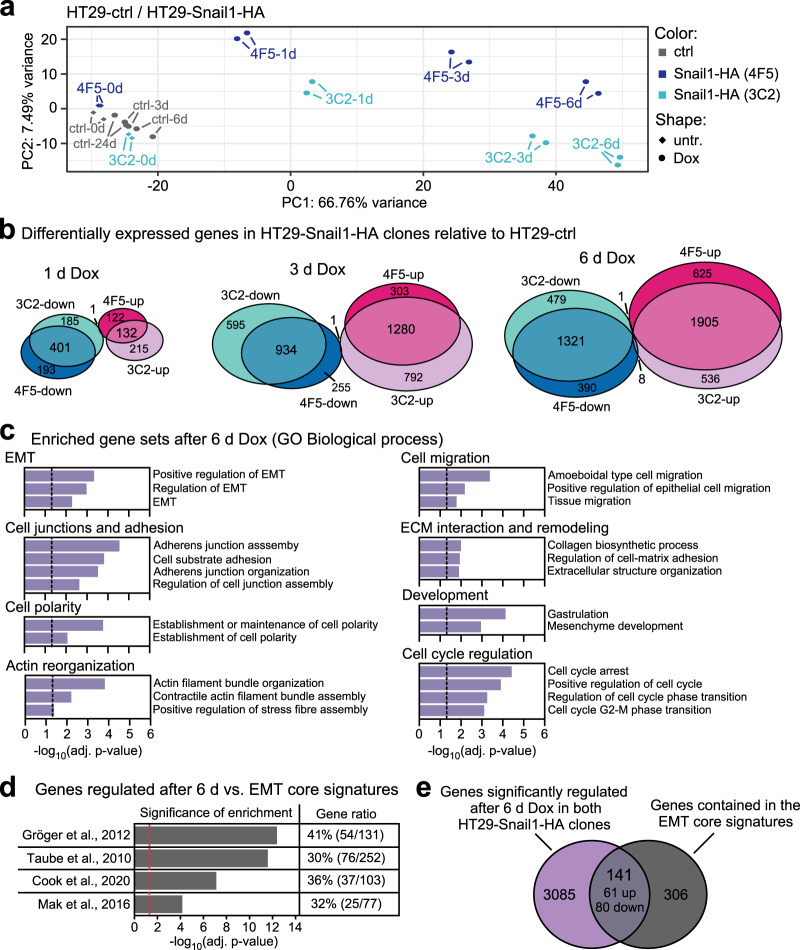
Transcriptomic analyses confirm that HT29-Snail1-HA cells undergo EMT. **a** Principal component analysis of microarray-derived transcriptome data from HT29-ctrl cells and HT29-Snail1-HA clones 4F5 and 3C2. Two biological replicates were analyzed for each condition. Cells were treated with Dox as indicated. **b** Venn diagrams indicating the numbers of differentially expressed genes (DEGs) in HT29-Snail1-HA clones 4F5 and 3C2 relative to HT29-ctrl cells after treatment with Dox for the indicated time spans. Genes are separated into downregulated (blue) and upregulated (magenta) genes. A cutoff of adjusted *p*-value < 0.05 was used to determine DEGs. Detailed results of differential gene expression analyses are listed in Table S1. **c** Gene set enrichment analysis determining significantly enriched gene sets among the genes upregulated in both HT29-Snail1-HA clones after 6 d of Dox treatment. The Gene Ontology gene set database “Biological process” was used. Vertical dotted lines indicate the applied threshold of adjusted (adj.) *p*-value < 0.05. Gene sets are grouped according to their association with typical traits of EMT. Only a selection of significantly enriched terms is shown. Detailed results of the analyses, also for downregulated genes, can be found in Table S2. **d** Gene set enrichment analysis to assess enrichment of EMT core signatures in the 3226 genes that were significantly deregulated in the same direction after 6 d of Dox treatment in the both HT29-Snail1-HA clones. EMT core gene signatures were derived from the publications indicated on the left. The vertical dotted line indicates the applied threshold of adj. *p*-value < 0.05. The share of genes from the respective core signatures that are significantly regulated in HT29-Snail1-HA is given on the right (Gene ratio). Detailed results of the analysis can be found in Table S3. **e** Venn diagram illustrating the overlap between genes that were significantly deregulated in the same direction in both HT29-Snail1-HA clones after 6 d of Dox treatment and 447 genes contained in at least one of the EMT core signatures. See also Table S4.

Having shown that selected EMT marker genes can be regulated in *SMAD4*^mut^ cells, we characterized the gene regulatory events evoked by Snail1-HA in HT29 cells at a global level by performing microarray-based transcriptomic experiments. Principal component analysis showed a high degree of similarity among biological replicates and demonstrated that the transcriptomes of Dox-treated HT29-Snail1-HA clones gradually diverged from those of uninduced cells over time (Fig. 4a). Next, we determined the differentially expressed genes (DEGs) after Snail1-HA induction (Fig. 4b, Table S1). We observed a quantitative increase in the number of regulated genes over time, with an initial preponderance of downregulation of gene expression that might be explained by the mainly repressive activities of Snail1-HA. When comparing DEGs in the HT29-Snail1-HA clones 4F5 and 3C2, clonal variability was limited and steadily decreased. Importantly, only 9 out of a total of 5265 DEGs were regulated in opposite directions in the two clones. This indicates a high degree of similarity between the transcriptomic changes in both clones, differing mainly in magnitude rather than in nature. To further analyze the DEGs, we focused on genes that were upregulated in both clones after 6 d of Dox treatment. Gene set enrichment analysis (GSEA) revealed that these genes were significantly enriched for gene sets associated with multiple well-characterized traits of EMT such as regulation of cell polarity and migration, reorganization of the actin cytoskeleton, modification of the extracellular matrix, and changes in cell cycle control (Fig. 4c, Table S2). Additionally, when we compared all 3226 DEGs to four signatures of genes that are broadly regulated during EMT processes [20, 22–24], we found that high percentages of the components of all four EMT gene signatures were significantly enriched among the DEGs from HT29-Snail1-HA cells (Fig. 4d, Table S3). In total, out of the 447 genes resulting from compilation of the four EMT core signatures, 141 genes were also regulated in HT29-Snail1-HA cells (Fig. 4e, Table S4). Taken together, transcriptomic analyses demonstrated closely related transcriptional responses of the two HT29 clones following Snail1-HA induction and confirmed the manifestation of EMT in HT29-Snail1-HA cells at a global transcriptional level.

### Plasticity of EMT gene regulatory mechanisms

**Fig. 5:**
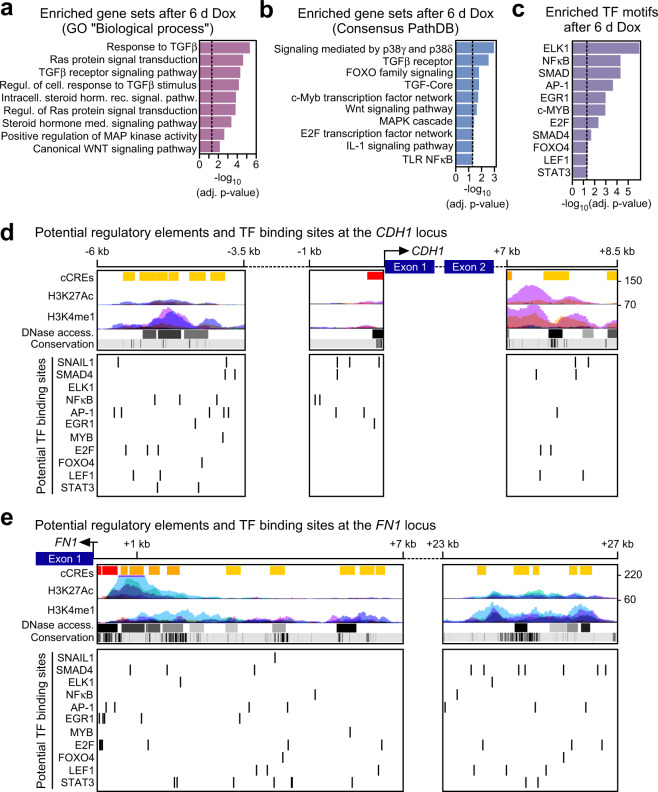
Candidate signal transduction pathways and transcription factors substituting for SMAD4 in the regulation of EMT-associated genes downstream of SNAIL1. **a** Gene set enrichment analysis determining significantly GO terms among the genes upregulated in both HT29-Snail1-HA clones after 6 d of Dox treatment. The Gene Ontology gene set database “Biological process” was used. Vertical dotted lines indicate the applied threshold of adjusted (adj.) *p*-value < 0.05. Detailed results can be found in Tables S2. **b**, **c** Gene set enrichment analyses determining significantly enriched gene sets in the 3226 genes that were deregulated in the same direction after 6 d of Dox treatment in both HT29-Snail1-HA clones. The ConsensusPathDB database (**b**), and the ‘Transcription factor targets’ collection of gene sets from the MSigDB database (**c**) were used for the analyses. Vertical dotted lines indicate the applied threshold of adjusted (adj.) *p-*value < 0.05. Only a selection of significantly enriched terms is shown. Detailed results can be found in Tables S5, S6, and S7. **d**, **e** Identification of potential regulatory elements and TF binding sites at the *CDH1* (**d**) and *FN1* (**e**) loci. Gene structures are shown on top. Dotted lines and exons are not drawn to scale. Below, corresponding snapshots of the UCSC genome browser are depicted showing the locations of candidate cis-regulatory elements (cCREs), H3K27Ac and H3K4me1 histone marks in 7 cell lines, and DNase I accessibility (access.) peak clusters from 95 cell lines all of which were derived from ENCODE. Numbers on the right denote vertical viewing range settings. An additional bar denotes sequence conservation across 100 species of vertebrates. Black lines indicate high conservation. At the bottom, locations of potential binding sites for the TFs identified in **c** are shown as vertical lines. To identify AP-1 binding sites all potential dimers of Fos and Jun family members and their predicted binding motifs were considered.

**Fig. 6:**
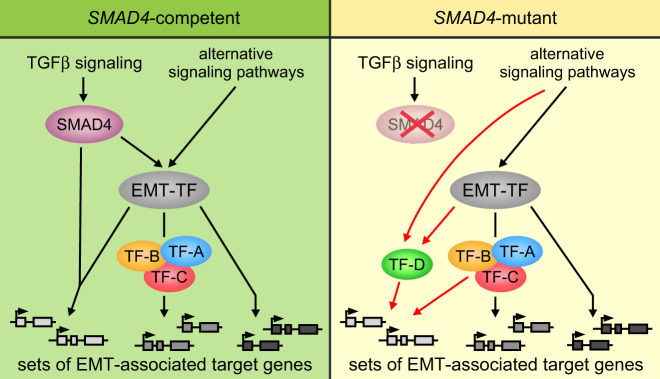
Model illustrating the proposed context-dependent roles of SMAD4 in the induction and execution of EMT. In *SMAD4*-competent backgrounds, canonical TGFβ signaling mediated by SMAD4, but also various alternative pathways, can trigger EMT by inducing expression of EMT-TFs. Following their induction, EMT-TFs execute EMT by regulating EMT-associated genes directly, as well as indirectly through changing the activity of other TFs (TFs A/B/C). This includes the cooperation of EMT-TFs with SMAD4 to regulate a subset of target genes. In *SMAD4*-mutant systems, despite the defect in the TGFβ pathway, EMT-TF expression can still be induced by alternative signaling pathways and direct and indirect regulation of EMT-associated genes by EMT-TFs and non-SMAD4 TFs can proceed normally. However, the missing contribution of SMAD4 to EMT-associated gene regulation has to be compensated for (red arrows). This could occur by non-SMAD4 TFs that are also employed in *SMAD4*-competent systems (TFs-A/B/C), or by factors whose implication in EMT execution is specific to *SMAD4*-mutant contexts (TF-D). By this, TGFβ-activated SMAD4 can contribute to EMT-TF induction and EMT-associated gene regulation in some instances of EMT, but it is not obligatory for EMT to occur.

The investigations described so far showed that Snail1-HA triggered a transcriptional response in *SMAD4*^mut^ cells which was highly similar to gene expression profiles associated with bona fide EMT processes. To get insight into the mechanistic basis of SMAD4-independent EMT execution, we performed further bioinformatic analyses of our transcriptome data. Surprisingly, despite proven pathway inactivity and *SMAD4*^mut^ status, GSEA pointed towards TGFβ signaling and SMAD4 as underlying the Snail1-HA-induced transcriptional effects in HT29 cells (Fig. 5a–c, Tables S2, S5, S6, S7). This suggested to us that significant parts of the genetic program characterizing EMT can be driven by canonical TGFβ signaling but also by other gene regulatory cascades. Candidate pathways and TF networks for alternative regulators of EMT-associated gene expression were likewise identified by GSEA. They include MAPK, WNT, and cytokine signaling, as well as MYB, E2F, and FOXO proteins (Fig. 5a–c). Consistent with the possibility that variable TF collectives facilitate EMT-associated gene expression, the regulatory regions of *CDH1* and *FN1*, two key players in EMT and among the DEGs in HT29-Snail1-HA cells, feature potential DNA binding motifs for SMAD proteins but also for ELK1, AP-1, EGR1, LEF1, NFκB, and STAT3, which are activated by MAPK, WNT, and cytokine signaling, respectively, as well as for MYB, E2F and FOXO4 (Fig. 5d, e). Notably, these alternative effectors of Snail1-HA-induced EMT were already implicated in EMT before [19, 30–38]. Accordingly, we envision that in *SMAD4*^mut^ backgrounds, one or several alternative TFs take over the transcriptional control functions of SMAD4 during EMT. A hypothetical model for the proposed plasticity of EMT gene regulatory mechanisms is depicted in Fig. 6.

### Analyses of human tumor transcriptomes indicate that *SMAD4* mutations do not preclude EMT execution in vivo

**Fig. 7:**
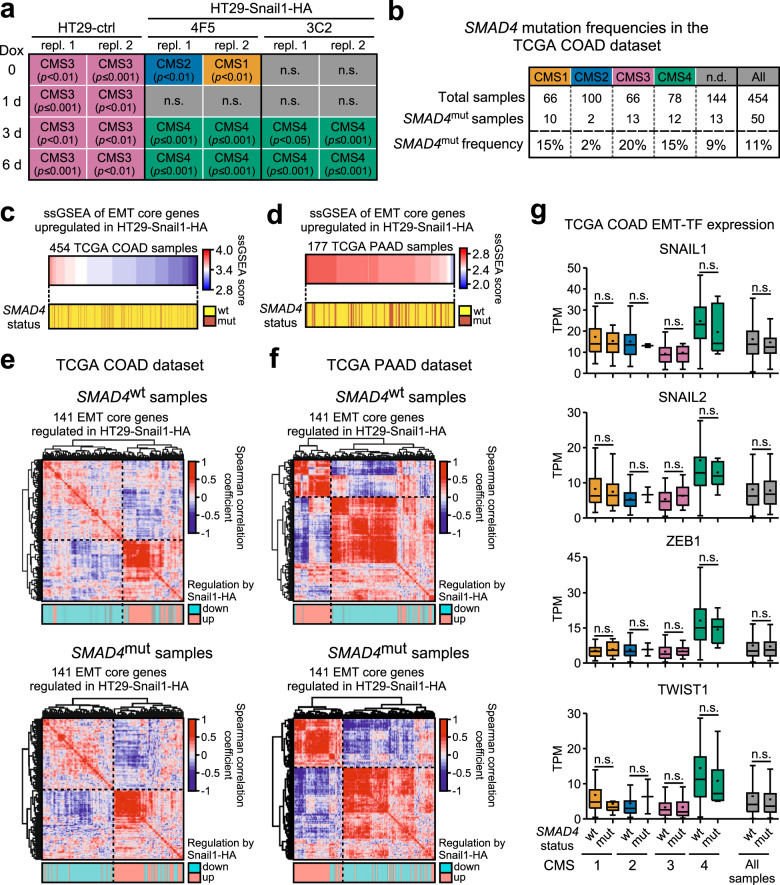
*SMAD4* mutations do not preclude an EMT-like transcriptome in human tumors in vivo. **a** Assignment of the HT29-ctrl and HT29-Snail1-HA transcriptome samples to consensus molecular subtypes (CMS) of colorectal cancer. For each condition, both replicates (repl.) are plotted individually. n.s.: no significant assignment to one of the four CMS. **b** *SMAD4* mutation frequencies in colon adenocarcinoma (COAD) samples from The Cancer Genome Atlas (TCGA) database. Numbers of total samples and *SMAD4*^mut^ frequencies for each CMS as well as in all samples in the dataset (All) are listed. n.d.: no significant CMS classification possible. **c, d** Single-sample gene set enrichment analysis (ssGSEA) of COAD (**c**) and pancreatic adenocarcinoma (PAAD, (**d**)) samples from the TCGA database. Scores for ssGSEA were calculated for each sample based on enrichment of the 61 genes that were upregulated in both HT29-Snail1-HA clones after 6 d of Dox treatment and are contained in at least one of the EMT core signatures (see Fig. 4d, e). The samples are ordered horizontally according to their ssGSEA score. The yellow/brown color bar below indicates the *SMAD4* status of each sample. **e, f** Correlation heatmaps displaying the mutual correlation of expression levels of the 141 genes that are significantly regulated in both HT29-Snail1-HA clones after 6 d of Dox treatment and are contained in at least one of the EMT core signatures (see Fig. 4d, e). Tumor transcriptomes were obtained from the TCGA database and samples were divided into two groups according to their *SMAD4* mutation status. For **e** COAD and for **f** PAAD samples were used. Clustering of genes was achieved by unsupervised hierarchical clustering based on the Euclidean distance. Dotted lines highlight the boundaries between the two principal gene clusters obtained in each case. Turquoise/pink color bars below each heatmap indicate whether a gene is up- or downregulated in HT29-Snail1-HA cells. **g** Expression levels of EMT transcription factor genes in COAD samples from the TCGA database. Samples were grouped by their CMS and further divided based on their *SMAD4* mutation status. Sample numbers for each condition are listed in **b**. Dots in the box plots indicate average values. Whisker lengths are based on the Tukey method. Outliers are not plotted but were considered for calculating significance. Significance was determined by differential gene expression analysis with the limma package in R/Bioconductor; n.s.: not significant (adj. *p*-value ≥ 0.05). TPM: transcripts per million.

Next, we assessed whether our in vitro findings could be representative of processes that occur in human carcinogenesis. For this, we investigated the potential semblance of HT29-ctrl and HT29-Snail1-HA cell transcriptomes to gene expression profiles of the CRC CMS classification [25]. While the HT29-ctrl cells were assigned to CMS3 irrespective of Dox treatment, the uninduced HT29-Snail1-HA clones could not be unambiguously associated with a specific subtype (Fig. 7a). This ambiguity is in accordance with reports that the HT29 cell line does not robustly associate with a specific CMS [39]. Strikingly, though, after treatment with Dox, the transcriptomes of both HT29-Snail1-HA clones consistently adopted features of CMS4 which comprises tumors with evidence for EMT and with the worst prognosis [25]. Thus, the transcriptomic changes observed in HT29-Snail1-HA cells in vitro resemble those that are observed during EMT in CRC in vivo.

Next, we examined the frequency of *SMAD4* mutations across CMS samples, assuming that they should be less frequent in CMS4 tumors if the *SMAD4*^mut^ status precluded EMT. However, using The Cancer Genome Atlas (TCGA) database of colon adenocarcinoma (COAD) transcriptomes, we found that *SMAD4*^mut^ tumors are not underrepresented among CMS4 samples when compared to other subtypes or all tumors in the database (Fig. 7b), raising the possibility that SMAD4 is also not obligatory for EMT in vivo.

To further corroborate SMAD4-independence of mesenchymal gene expression by a second approach to define mesenchymal tumors that was not related to the CMS classification, we performed single-sample GSEA (ssGSEA). This method enables to assess whether constituents of a particular gene set are overrepresented among the most highly or most lowly expressed genes of a given transcriptome, resulting in a ssGSEA score that is assigned to each sample. As a quantitative measure for a mesenchymal state, we used as input gene set the 61 genes that were upregulated by Snail1-HA in HT29 cells and were contained in at least one of the EMT core signatures (see Fig. 4e). When we applied ssGSEA to 454 TCGA COAD samples and then ordered these according to their ssGSEA scores from highest to lowest, that is from most to least mesenchymal, there was no underrepresentation of *SMAD4*^mut^ samples in the samples with the highest ssGSEA score (Fig. 7c). Rather, *SMAD4*^mut^ samples were distributed evenly along the spectrum. When repeating the analysis with pancreatic adenocarcinoma (PAAD) samples that also frequently show *SMAD4* mutations, a similar random distribution was observed (Fig. 7d). Thus, using two different approaches to identify tumors with strong evidence for EMT, we could show that *SMAD4* mutations are not less frequent in mesenchymal tumors of the colon and the pancreas.

To further interrogate the relationship between *SMAD4* status and EMT-related gene expression, we performed mutual correlation analyses of gene expression based on the TCGA COAD and PAAD samples and the 141 EMT core genes that were regulated by Snail1-HA in HT29 cells. In both, COAD and PAAD *SMAD4*^wt^ samples, these genes separated into two clusters, which were predominantly formed by the up- and downregulated subgroups of the 141 EMT core genes (Fig. 7e, f). Consistent with their direction of regulation in the HT29 cell background, expression of genes within each of the two clusters was mostly positively correlated whereas the two clusters exhibited anti-correlated expression profiles. Importantly, this anti-correlation was also apparent in *SMAD4*^mut^ samples.

In order for our CRC cell line models of Snail1-HA-inducible, SMAD4-independent EMT execution to be applicable to human cancer, not only would the EMT-associated gene expression be expected to be independent of SMAD4, but also the expression of EMT-TFs should be increased in mesenchymal tumor samples regardless of *SMAD4* status. To test this, we stratified TCGA COAD samples according to CMS and further subdivided each group by *SMAD4* status. We then extracted expression values for EMT-TFs from all samples and compared them with respect to CMS classification and SMAD4 status (Fig. 7g). Overall, EMT-TF expression in CMS4 samples appeared to be higher than in CMS1-3, which is consistent with the more mesenchymal character of CMS4 tumors and the EMT-inducing capacity of EMT-TFs. Yet, while their expression was not affected by *SMAD4* status in CMS1-3, EMT-TF expression in CMS4 samples tended to be lower in *SMAD4*^mut^ tumors compared to *SMAD4*^wt^, albeit this did not reach statistical significance. Importantly, EMT-TF expression in *SMAD4*^mut^ CMS4 samples was still markedly higher than in samples from the other CRC subtypes. A possible explanation for these observations could be that *SMAD4* mutations lead to inactivation of the TGFβ pathway and therefore disable an inducer of EMT-TFs in some cases. Evidence for TGFβ signaling possibly being active and thus upregulating EMT-TFs in some cases is provided by higher expression of TGFβ ligand and receptor genes again in CMS4 samples (Fig. S7), which is entirely expected since indication of TGFβ pathway activation is a defining feature of CMS4 [25]. This notwithstanding, elevated levels of EMT-TFs also in *SMAD4*^mut^ samples argue that alternative signal transduction pathways with the capacity to induce or maintain high expression of EMT-TFs seemingly operate in CMS4 tumors. Furthermore, not only EMT-TF expression but also CRC prognosis appeared to be unaffected by *SMAD4* status since the *SMAD4*^mut^ condition did not confer survival advantages or disadvantages to cases with high or low *SNAIL1* expression. Likewise, survival of CRC patients with CMS4 tumors was the same for *SMAD4*^wt^ and *SMAD4*^mut^ cases (Fig. S8). Apparently, the EMT or EMT-like state of poor prognosis CMS4 cancers does not require SMAD4. These observations are in keeping with the results of our cell culture studies where Snail1-HA could induce EMT and, hence, a more malignant phenotype in SMAD4-deficient cells. In conclusion, our investigations based on human tumor transcriptomes revealed that *SMAD4* mutations do not preclude elevated expression of EMT-TFs and the occurrence of gene expression patterns indicative of EMT in colorectal and pancreatic tumors. Thus, mesenchymal phenotypes can exist in vivo despite the absence of SMAD4, arguing that EMT may occur regardless of the *SMAD4* status in human tumors from different entities.

## Discussion

Here, we demonstrate that CRC cell lines can undergo EMT independently of SMAD4 expression and TGFβ/BMP type I receptor activity after induction of the EMT-TF SNAIL1. Correspondingly, analyses of human tumor transcriptomes showed that *SMAD4* mutations were neither underrepresented, nor did they abrogate elevated expression of EMT-TFs in tumor samples exhibiting features of EMT. Thus, our results establish that EMT is not categorically precluded in *SMAD4*^mut^ tumors.

SMAD complexes activated by TGFβ/BMP growth factors were previously described to be crucial for the gene regulatory cascades underlying EMT both upstream and downstream of EMT-TFs [14, 15, 18, 19, 40], which is in stark contrast to our current findings. For example, SMAD complexes were shown to mediate the Snail1-induced downregulation of *CDH1*, *OCLN* and *CLDN3* in mouse mammary epithelial cells [18], whereas downregulation of the same three genes occurred independently of SMAD4 in our model systems. Likewise, ligand/receptor-activated BMP pathway activity and SMAD4 were critical for Snail1-HA-induced EMT execution in *SMAD4*^wt^ CRC cells [19], but turned out to be entirely dispensable in the *SMAD4*^mut^ HT29 and SW403 cells. To conjoin these observations, we propose that a sizeable fraction of the genetic program characteristic for EMT can be driven in a manner dependent on TGFβ signaling and SMAD4-containing protein complexes but also by alternative regulatory cascades. Support for this postulated plasticity in transcriptional control of EMT processes is provided by experimental evidence for alternative regulation of *FN1* expression by LEF1 and by NFκB [36, 41], and by recent findings demonstrating considerable context-dependence and heterogeneity among gene regulatory networks operating during EMT in different systems [14, 20, 21].

Irrespective of the mechanistic plasticity of EMT-associated gene regulation, implementation of EMT in *SMAD4*^mut^ backgrounds would require SMAD4-independent induction of EMT-TFs in the first place. Compliant with this idea, our analyses of tumor transcriptomes showed elevated EMT-TF expression also in *SMAD4*^mut^ mesenchymal tumors. Thus, EMT-TF expression appears to be regulated by alternative signaling pathways in certain cancer contexts. This is plausible considering that for example expression of SNAIL1 and ZEB1 clearly does not solely depend on TGFβ pathway activity and can be induced by WNT, MAPK, and several other signaling cascades [10, 42, 43].

Our results further demonstrate that EMT in HT29-Snail1-HA cells proceeds irrespective of TGFβ/BMP receptor activities, which rules out a contribution not only of canonical but also of non-canonical TGFβ superfamily signaling pathways. This suggests that besides the dysfunction of SMAD4, also mutations in other components of TGFβ superfamily signaling pathways, like the frequent mutations in *TGFBR2* and *ACVR2A* in microsatellite-instable tumors [5], would likewise not exclude the occurrence of EMT. In support of this, we and others previously reported SNAIL1-induced EMT in *TGFBR2*^mut^ CRC cell lines [44–46]. Notably, however, other studies suggested that *SMAD4*^mut^ cancer cells might benefit from SMAD4-independent TGFβ/BMP receptor-driven signaling to undergo EMT-like changes [47, 48], arguing for further complexity and plasticity among the routes to EMT in *SMAD4*^mut^ tumors.

While we could not confirm this for our TCGA CRC data set, clinical observations indicated that *SMAD4*^mut^ tumors confer poor prognosis for the survival of patients with different cancer types [7, 8, 49, 50]. In light of our findings, a possible explanation for this could be that *SMAD4*^mut^ tumors on the one hand escape the tumor-suppressive function of TGFβ signaling, while on the other hand they retain the potential to become metastatic and therapy-resistant by undergoing SMAD4-independent EMT. However, other groups provided alternative explanations for the increased malignancy of *SMAD4*^mut^ tumors such as enhanced chemoresistance [51] and increased recruitment of myeloid-derived suppressor cells facilitating tumor cell invasion [52]. Future studies will need to address whether these mechanisms operate alternatively to SMAD4-independent EMT or in conjunction with it.

Although our investigations of human tumor transcriptome data suggest that EMT-TF induction and EMT execution are not impeded by the *SMAD4*^mut^ state, a limitation of these analyses is that they are unable to resolve the temporal order of events. Thus, in some tumors, *SMAD4* mutations might have been acquired only after EMT had been implemented. However, the observation that the transcriptomes of *SMAD4*^mut^ HT29 cells upon Snail1-HA expression assimilate characteristics of EMT-associated CMS4 tumors over time, may serve as proof-of-principle arguing that the mesenchymal gene expression patterns observed in vivo might also be established after the acquisition of *SMAD4* mutations. Nonetheless, it remains subject to further studies to precisely determine the order of events and the frequency of EMT post *SMAD4* inactivation in vivo.

In summary, we establish that EMT can be implemented independently of SMAD4 in cancer cells. Thereby, our results corroborate substantial plasticity in the pathways orchestrating EMT processes in different settings. Specifically, our findings are relevant for the evaluation of *SMAD4*^mut^ tumors in the context of EMT and its potential therapeutic targeting and add to our understanding of mechanisms underlying their capacity to become invasive and to metastasize.

## Materials and methods

### Cell culture

Human colorectal cancer cell lines were obtained from different sources (HCT116: ATCC® CCL-247 from the MPI-IE Freiburg; LS174T: #300392 from Cell Line Service [CLS], Eppelheim, Germany; HT29: #300215 from CLS; SW403: CCL-230 from ATCC®, Manassas, Virginia, USA). All parental cell lines and the HT29-ctrl and SW403-ctrl cells, as well as the HT29-Snail1-HA (4F5, 3C2) and SW403-Snail1-HA (1B10, 1F7) single cell clones were authenticated by SNP-profiling at Multiplexion Inc. (Friedrichshafen, Germany). Cells were cultured under previously described conditions [19]. All cells were screened on a regular basis for mycoplasma infection using the MycoSensor PCR assay kit (#302109; Agilent, Santa Clara, California, USA). When required, cells were treated with doxycycline (Dox) (D-9891; Sigma-Aldrich, St. Louis, Missouri, USA) at a concentration of 1 µg ml^−1^. Where indicated, LDN193189 (#1062368-24-4; Cayman Chemical Ann Arbor, Michigan, USA) was applied at 50 nM and SB431542 (#S1067; Selleckchem Houston, Texas, USA) at 10 µM. DMSO (D-5879; Sigma-Aldrich) amounts used as control treatments were equal to the volumes administered when applying both receptor inhibitors in combination. For treatment of cells with growth factors, BMP4 (#120-05ET; Peprotech; Cranbury, New Jersey, USA) was used at 100 ng ml^−1^ and TGFβ1 (#100-21; Peprotech) at 5 ng ml^−1^, respectively. Media of treated cells were refreshed every second day.

### Generation of stable cell lines

HT29-Snail1-HA and SW403-Snail1-HA parental cells were generated by lentiviral transduction with the previously described pMuLE_LentiDest_Xtight-Snail1-HA_SV40-rtTA2s-M2_eGFP-PuroR vector. This vector includes cassettes for constitutive expression of eGFP, a puromycin resistance gene, and the reverse tetracycline-dependent transactivator rtTA2. In addition, it harbors a Dox-inducible expression cassette for Snail1-HA [36]. HT29-ctrl and SW403-ctrl cells were generated using a similar plasmid lacking the Snail1-HA coding sequence. Because the Dox response of the initially obtained HT29-Snail1-HA and SW403-Snail1-HA mixed cell populations was heterogeneous even after complete puromycin selection, eGFP^+^ single cells were sorted by FACS and distributed into 96-well plates. After single-cell-derived clones had grown to sufficient density, plates were carbon-copied by splitting, and the clones were screened by microscopy for phenotypic changes after addition of Dox to the copy plate for 6 d. For each cell line, eight clones that strongly and homogeneously changed morphology, as well as two non-responding clones were then expanded from the original, untreated 96-well plate and further analyzed.

### Analyses of gene expression at mRNA level

For targeted analyses of gene expression at mRNA level, the PeqGOLD total RNA kit (#732–2871; Peqlab/VWR Life Science Bruchsal, Germany) for RNA extraction and the qScript™ Flex cDNA Kit (#95049; Quantabio Beverly, MA, USA) for cDNA preparation were used. qRT-PCR reactions were carried out on a CFX384 Touch Real-Time PCR Detection System (BioRad Laboratories, Hercules, CA, USA) using PerfeCTa® SYBR® GreenSuperMix (#95054; Quantabio). Relative gene expression was calculated using the 2-ΔCt method after normalizing C_t_-values to those of the housekeeping gene *GAPDH*. All oligonucleotides used are listed in Table S8. Global transcriptome analysis using microarrays were performed as previously described [19]. Differentially expressed genes (DEGs) were determined via the limma package from R/Bioconductor [53]. Genes with an adjusted *p*-value < 0.05 were considered differentially expressed (Benjamini-Hochberg correction). Microarray data are available in the Gene Expression Omnibus (GEO) repository under the accession number GSE169735. To extract differentially expressed TF genes from the microarray results, human TFs were downloaded from The Human Transcription Factors website (http://humantfs.ccbr.utoronto.ca/download.php; accessed on April 27, 2020). Genes annotated as TF were retained and compared with the 3226 DEGs common to HT29-Snail1-HA clones 3C2 and 4F5 after 144 h. TFs were considered to be regulated by Snail1-HA if the adjusted *p*-value was <0.05 and the absolute log2FC was >0.5. As the 3226 DEGs were obtained by the intersection of the DEGs between clones 3C2 and 4F5 (having the same log2FC orientation), we calculated the average log2FC value and used this as a selection criterion for the TFs.

### Gene set enrichment analyses

Fisher’s exact test was used to determine enriched gene sets from the Gene Ontology database collection “Biological process” [54], the Transcription Factor Targets database [55], and the Consensus database [56] based on the lists of DEGs in HT29-Snail1-HA versus HT29-ctrl cells. Significance threshold was routinely set to adjusted *p*-value < 0.05. To calculate single sample gene set enrichment scores for each TCGA tumor sample, the GSVA package from R/Bioconductor (version 1.32) was used [57].

### Processing of TCGA data sets

The TCGA database was accessed on February 10th, 2020 via the TCGAbiolinks package [58] and 521 COAD RNAseq V2 data sets were downloaded for analyses. From these samples, 67 were filtered out due to non-colon primary site, duplicate patient ID, or absent clinical information. The CMScaller package [59] was used to define the CMS of the TCGA COAD samples and of the transcriptomes from HT29 derivatives. The TCGA PAAD samples were accessed similarly on February 3rd, 2020 and 182 PAAD RNAseq V2 data sets were downloaded. From these, 5 were filtered out due to non-pancreas primary site. The Spearman correlation coefficient was used to quantify the correlation scores of gene expression across tumor samples. The pheatmap package (version 1.0.12) [60] was used for representation of the results. To analyze the impact of *SMAD4* mutations on CRC patient survival, TCGA COAD samples were separated into two groups according to SNAIL1 expression. For this, the 50% of samples with the highest expression of SNAIL1 were placed in the high expression group while the remaining 50% were placed in the low expression group. Then, the groups were subdivided into *SMAD4*^wt^ and *SMAD4*^mut^ cohorts. Similarly, CMS4 samples were stratified according to their *SMAD4* mutation status. Survival curves were plotted by the Kaplan–Meier method using the survminer R package (https://cran.r-project.org/web/packages/survminer/index.html) and *p*-values were assessed based on logrank test.

### Immunoblotting and immunofluorescence stainings

Analyses of protein levels by SDS-PAGE and immunoblotting were performed using whole-cell lysates as previously described [61], with the exception that 100 µM sodium orthovanadate, 10 mM sodium fluoride, 1 mM PMSF, and 1% Phosphatase Inhibitor Cocktail solutions 2 and 3 (P-5726 and P-0044; Sigma-Aldrich) were additionally added to the lysis buffer. For all immunoblotting experiments a total of three independent biological replicates were carried out. Quantification of band intensities was performed using ImageJ (https://imagej.nih.gov/ij/docs/menus/analyze.html#gels). The primary antibodies used are listed in Table S9. Immunofluorescence staining were done as described [62] except that SW403 cell derivatives were plated on coverslips coated with 0.1 mg ml^−1^ poly-L-ornithine. Primary and secondary antibodies used are listed in Table S9. Images were acquired with a Zeiss AxioCam mounted on a Zeiss Axio Observer Z1 fluorescence microscope. For imaging and initial processing, the Zeiss AxioVision program SE64 was used. Upon assembly of images into a single file and software-mediated reduction of selected areas, if necessary, brightness, contrast, and color balance of midtones were adjusted (Canvas™, Canvas GFX, Inc., Fort Lauderdale, USA), whereby all panels from one series of stainings were treated identically.

### Transwell migration assays

For transwell migration assays, HT29 and SW403 cell derivatives were first seeded in 6-well plates and were left untreated or received Dox at 1 µg ml^−1^ for 5 d. Subsequently, cells were transferred to the upper chambers of transwell membrane inserts with 8 µm pore size (#353097; Corning; Corning, New York, USA) containing cell culture medium without FCS. Standard cell culture medium, with or without Dox, containing 10% FCS was added to the lower chamber, followed by 24 h of incubation allowing for cell migration across the insert membrane. Afterwards, cells that remained in the upper chamber were removed manually with cotton swabs. Cells on the lower surface of the membrane were fixed with 4% PFA and stained with 0.1% crystal violet solution for 10 min at room temperature. After extensive washing with distilled water, transwell inserts were allowed to dry and then imaged with a BZ-9000 microscope (Keyence Deutschland GmbH, Neu Isenburg, Germany). Cell migration was quantified using ImageJ by first creating a binary image and subsequently measuring the mean gray value of the transwell area.

### Spheroid invasion assays

Spheroid invasion assays were performed as previously described [44], except that Dox was applied at 1 µg ml^−1^ and pictures were taken nine days after embedding of spheroids into the collagen I matrix. Invasiveness was quantified by manually counting cells that were separated from the spheroid bodies using ImageJ. During quantification, the analyzing scientist was blinded to the identity of the cells used for the spheroid cultures. Viability of SW403 spheroids was assessed by testing their ability to reduce MTT to its purple formazan derivative. For this, after imaging individual spheroids, MTT solution (CT01-5; Sigma-Aldrich) was added to the media at a final concentration of 0.5 mg ml^−1^, followed by incubation for 2 h at 37 °C and subsequent top-view image acquisition on a document scanner.

### Sequence analysis of *SMAD4* exon 8

To analyze the sequence of *SMAD4* transcripts from LS174T and HT29-ctrl cells, and from HT29-Snail1-HA clones, parts of the *SMAD4* coding sequence were amplified by PCR from cDNA using a high-fidelity polymerase. The PCR products were then gel extracted and sent for Sanger sequencing at Microsynth AG (Balgach, Switzerland). Primer sequences are given in Table S8.

### Identification of potential regulatory elements and TF binding sites

The UCSC genome browser [63] was used for discovery and sequence analysis of potential regulatory elements at EMT-associated genes. Elements were identified by assessing locations of candidate cis-Regulatory Elements (cCREs), ChIP-Seq signals of H3K27Ac and H3K4me1, and DNase I Hypersensitivity peak clusters all of which were generated through the ENCODE consortium [64] and are contained in the track collection ‘integrated regulation’. Additionally, evolutionary sequence conservation was investigated using the integrated ‘Conservation 100 vertebrates by PhastCons’ tracks. Potential TF binding sites (TFBS) were identified using the UCSC genome browser tracks from the TFBS predictions in *homo sapiens* (hg38) for all TFBS profiles in the JASPAR CORE vertebrates collection (2020) using a track score cutoff of 400 which equals *p*-value < 0.0001 [65].

## Supplementary information

Supplementary Information Frey et al

Supplementary Table S1

Supplementary Table S2

Supplementary Table S3

Supplementary Table S4

Supplementary Table S5

Supplementary Table S6

Supplementary Table S7

Supplementary Table S8

Supplementary Table S9

## Data Availability

The microarray data were deposited in the Gene Expression Omnibus under the accession number GSE169735. All other data generated to back our results and conclusions are available from the corresponding author on reasonable request.

## Code availability

All code used will be made available upon request.
