# Supplementary material for: SMAD4 mutations do not preclude epithelial–mesenchymal transition in colorectal cancer
Source: Oncogene. 2021 Dec 3;41(6):824–37. doi: 10.1038/s41388-021-02128-2 (PMC8816731; doi:10.1038/s41388-021-02128-2)
Supplement: Supplementary file 1 — Supplementary Information Frey et al [file 41388_2021_2128_MOESM1_ESM.pdf]

Supplementary Information for

SMAD4 mutations do not preclude epithelial-mesenchymal transition in colorectal cancer

Patrick Frey, Antoine Devisme, Katja Rose, Monika Schrempp, Vivien Freihen, Geoffroy Andrieux, Melanie Boerries, and Andreas Hecht

This PDF file includes: Supplementary Figures 1 to 9

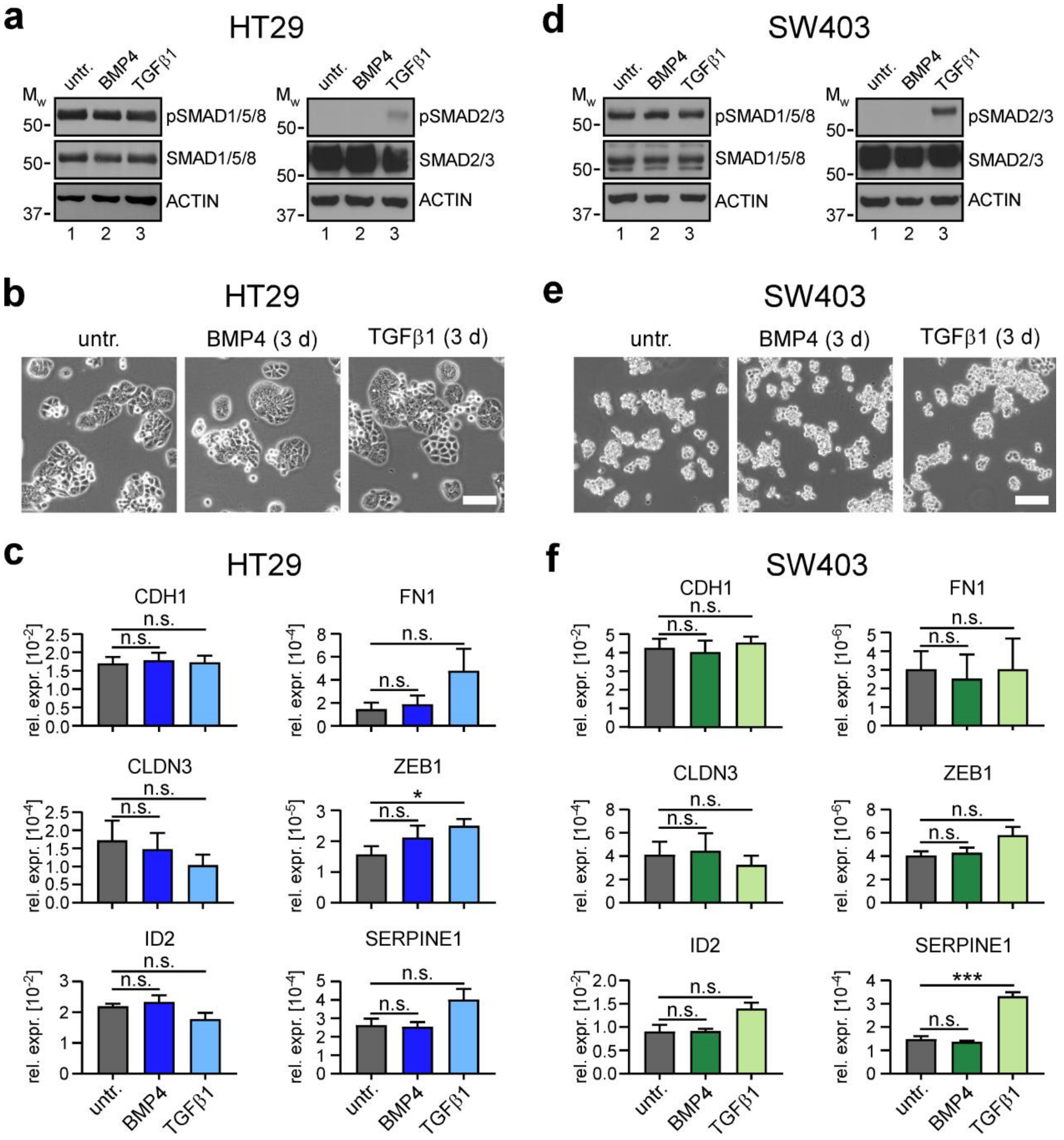

Supplementary Figure S1: Treatment with TGFβ superfamily ligands does not evoke EMT in *SMAD4*^{mut} CRC cell lines. (**a**) Analyses of protein expression by immunoblotting in HT29 cells untreated (untr.) or treated with 100 ng ml^{-1} BMP4 or 5 ng ml^{-1} TGFβ1 for 3 d as indicated. Positions of molecular weight (M_w) standards in kDa are indicated on the left. (**b**) Representative phase contrast images of HT29 cells treated with or without BMP4 or TGFβ1 as in (a). Scale bar: 100 µm. (**c**) Analyses of mRNA expression by qRT-PCR in HT29 cells treated with or without BMP4 or TGFβ1 as in (a). Relative gene expression (rel. expr.) was calculated by normalizing to the expression of *GAPDH*. Plotted is the mean + SEM; n = 4. Two-tailed student's t-test; *: p-value < 0.05. (**d**) Analyses of protein expression by immunoblotting in SW403 cells treated with or without BMP4 or TGFβ1 as in (a). Positions of molecular weight (M_w) standards are indicated on the left. (**e**) Representative phase contrast images of SW403 cells treated with or without BMP4 or TGFβ1 as in (a). Scale bar: 100 µm. (**f**) Analyses of mRNA expression by qRT-PCR in SW403 cells treated with or without BMP4 or TGFβ1 as in

(a). Relative gene expression (rel. expr.) was calculated by normalizing to the expression of *GAPDH*. Plotted is the mean + SEM; $n = 4$. Two-tailed student's t-test; ***: p-value < 0.001.

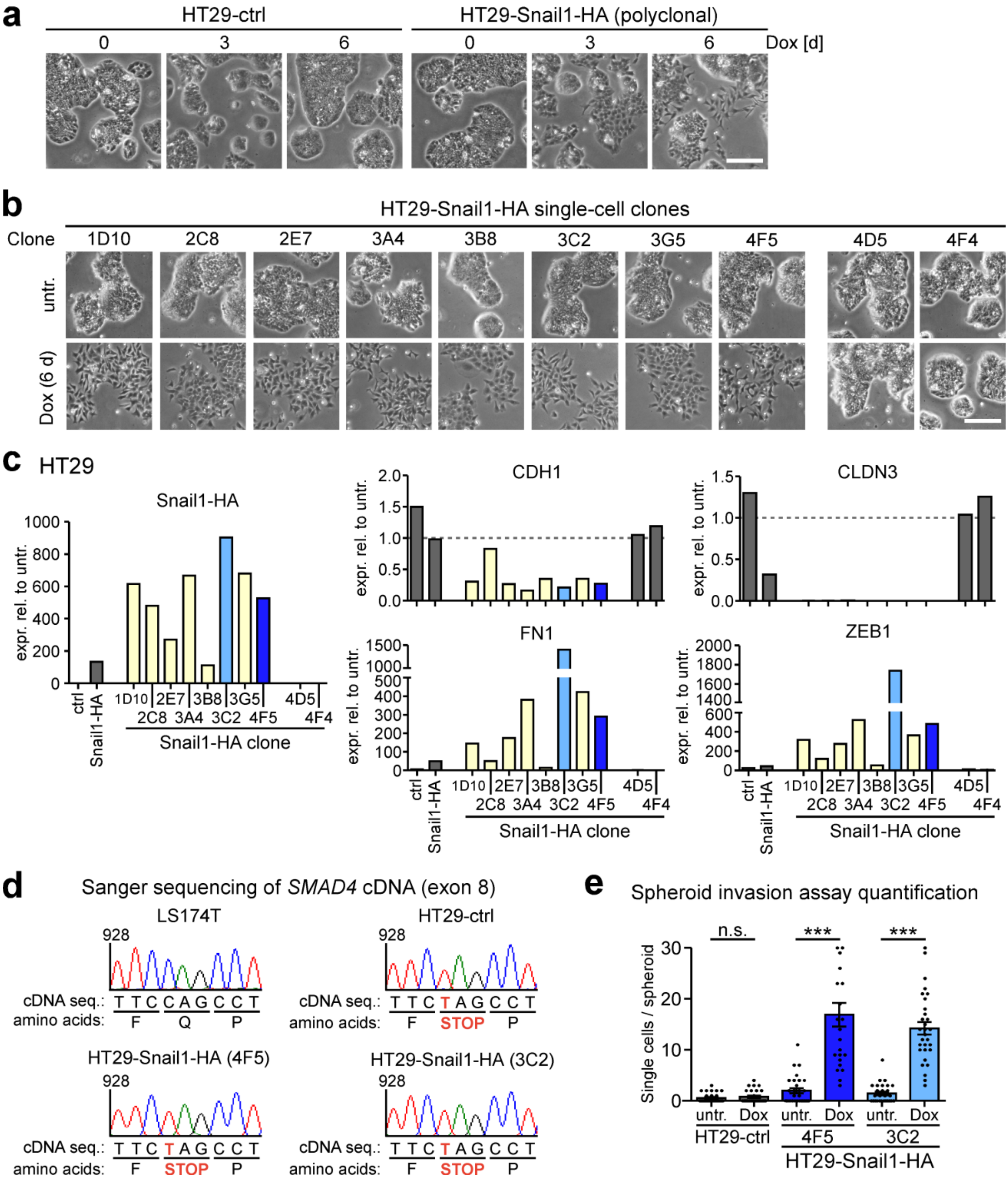

Supplementary Figure S2: Generation and characterization of HT29-Snail1-HA single-cell clones. (**a**) Representative phase contrast images of HT29-ctrl cells and the polyclonal HT29-Snail1-HA cell population treated with Dox as indicated. Scale bar: 200 µm. (**b**) Representative phase contrast images of ten selected HT29-Snail1-HA single-cell clones treated with Dox as indicated. Scale bar: 200 µm. (**c**) Analyses of mRNA expression by qRT-PCR in HT29-ctrl cells and the polyclonal HT29-Snail1-HA cell population as well as in ten selected HT29-Snail1-HA single-cell clones. Plotted is the gene expression in cells which had received Dox for 6 d relative to cells that had been left untreated (untr.). For each condition, relative gene expression was calculated by normalizing to the expression of *GAPDH*. Values of the HT29-Snail1-HA clones 4F5 and 3C2, which were chosen for further analyses, are highlighted in

blue. *n* = 1. (**d**) Representative electropherograms obtained by Sanger sequencing performed on *SMAD4* cDNA from LS174T cells, HT29-ctrl cells, or HT29-Snail1-HA clones 4F5 and 3C2. Sequences of nucleotides and the corresponding amino acids after translation are given on the bottom starting at position +928 from the canonical start codon in the *SMAD4* cDNA. *n* = 2. (**e**) Quantification of spheroid invasion assays with HT29-ctrl cells and HT29-Snail1-HA clones 4F5 and 3C2 by manual counting of single cells that were separated from the spheroid body. Representative spheroid images are shown in Figure 1f. A total number of ≥ 23 spheroids compiled from three biological replicates were quantified for each condition. Two-tailed Mann-Whitney U test; n.s.: not significant, ***: p-value < 0.001.

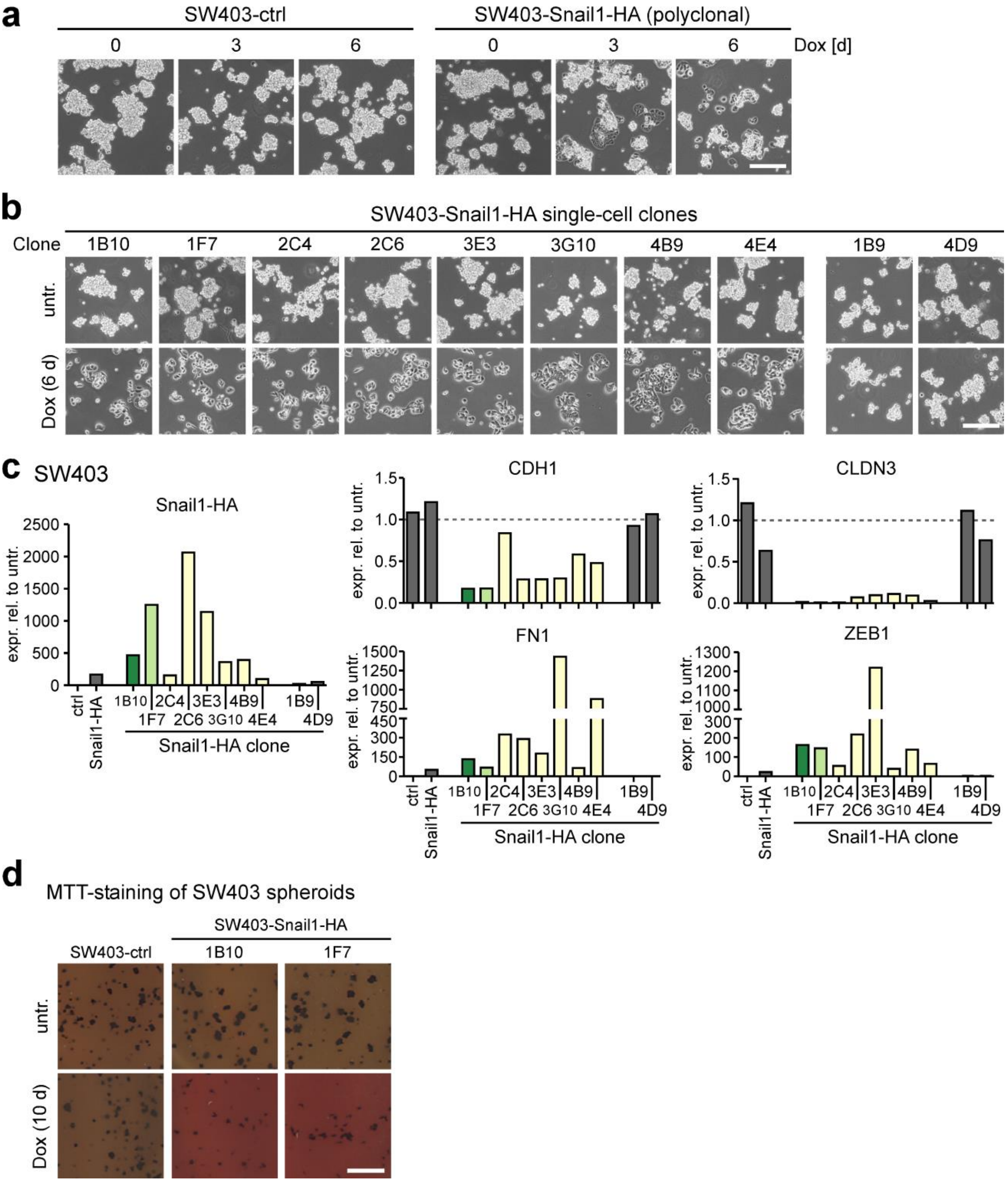

Supplementary Figure S3: Generation and characterization of SW403-Snail1-HA single-cell clones. (**a**) Representative phase contrast images of SW403-ctrl cells and the polyclonal SW403-Snail1-HA cell population treated with Dox as indicated. Scale bar: 200 µm. (**b**) Representative phase contrast images of ten selected SW403-Snail1-HA single-cell clones treated with Dox as indicated. Scale bar: 200 µm. (**c**) Analyses of mRNA expression by qRT-PCR in SW403-ctrl cells and the polyclonal SW403-Snail1-HA cell population as well as in ten selected SW403-Snail1-HA single-cell clones. Plotted is the gene expression in cells that received Dox for 6 d relative to cells that were left untreated (untr.). For each condition, relative gene expression was calculated by normalizing to the expression of *GAPDH*. Grey horizontal

dotted lines indicate the positions of relative expression = 1.0. Values of the SW403-Snail1-HA clones 1B10 and 1F7, which were chosen for further analyses, are highlighted in green. n = 1. (**d**) Representative top view pictures of spheroid populations from SW403-ctrl and SW403-Snail1-HA cells (clones 1B10, 1F7) stained with MTT solution. Viable cells take on a purple color due to reduction of the MTT dye. n = 2. Scale bar: 2 mm.

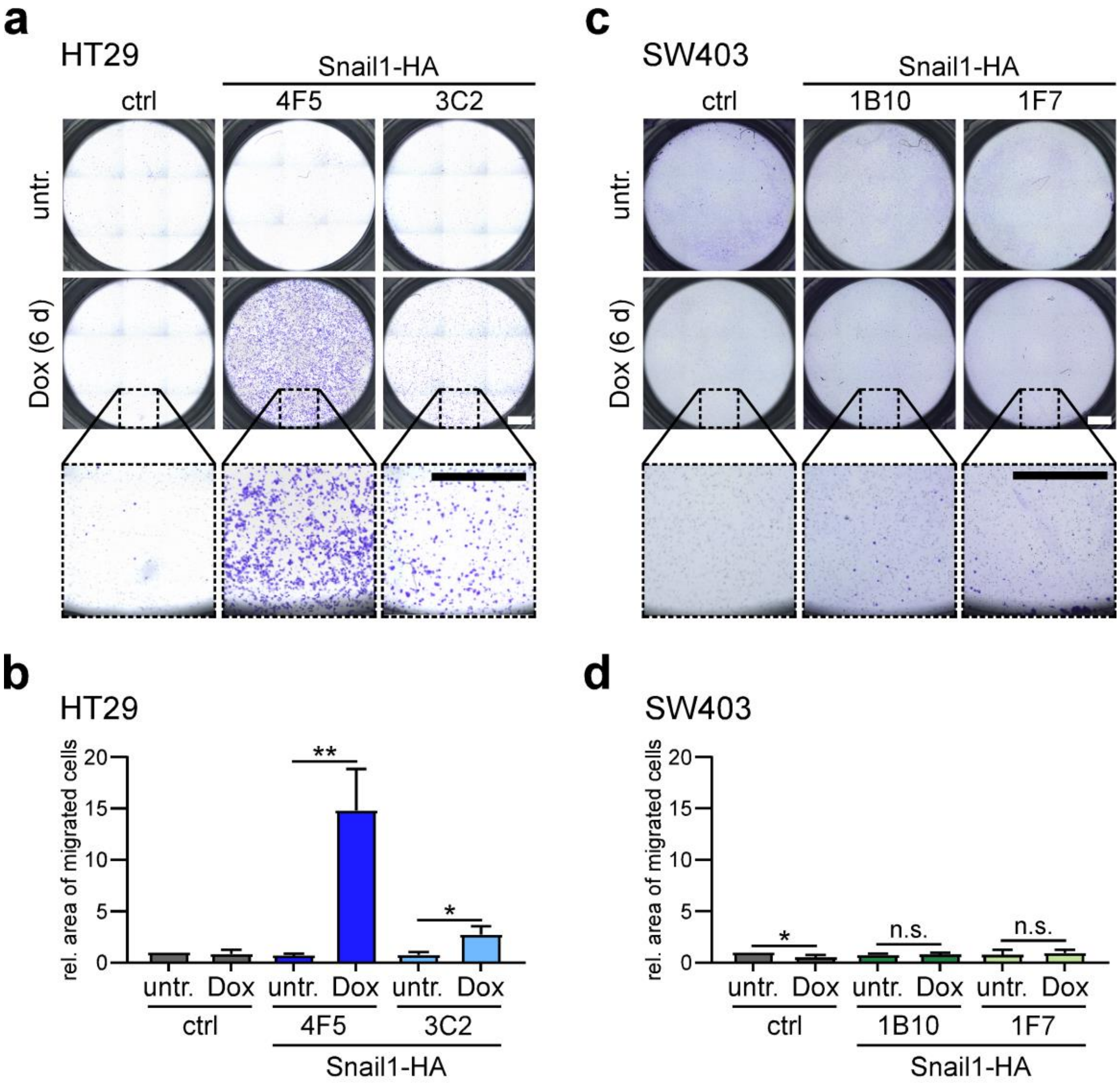

Supplementary Figure S4: Migratory capacities of HT29 and SW403 *SMAD4*^{mut} CRC cells after Snail1-HA induction. (**a**) Transwell migration assay of HT29-ctrl cells and HT29-Snail1-HA clones 4F5 and 3C2 that were treated with Dox as indicated. For each condition, one representative image of a transwell insert membrane stained with crystal violet is shown. Areas in dashed black boxes are additionally depicted in higher magnification below. Scale bars: 1 mm. (**b**) Quantification of cell migration in experiments as shown in (a). The area covered by cells on the lower part of the transwell inserts was determined for each condition and is shown relative to the value in untreated control cells. Plotted is the mean + SEM; $n = 3$. Two-tailed student's t-test; *: p-value < 0.05, **: p-value < 0.01. (**c**) Transwell migration assay of SW403-ctrl cells and SW403-Snail1-HA clones 1B10 and 1F7 that were treated with Dox as indicated. For each condition, one representative image of a transwell insert membrane stained with crystal violet is shown. Areas in dashed black boxes are additionally depicted in higher magnification below. Scale bars: 1 mm. (**d**) Quantification of cell migration in experiments as shown in (c). The area covered by cells on the lower part of the transwell inserts was determined for each condition and is shown relative to the value in untreated control cells. Plotted is the mean + SEM; $n = 3$. Two-tailed student's t-test; *: p-value < 0.05. (**a**-**d**) untr.: untreated.

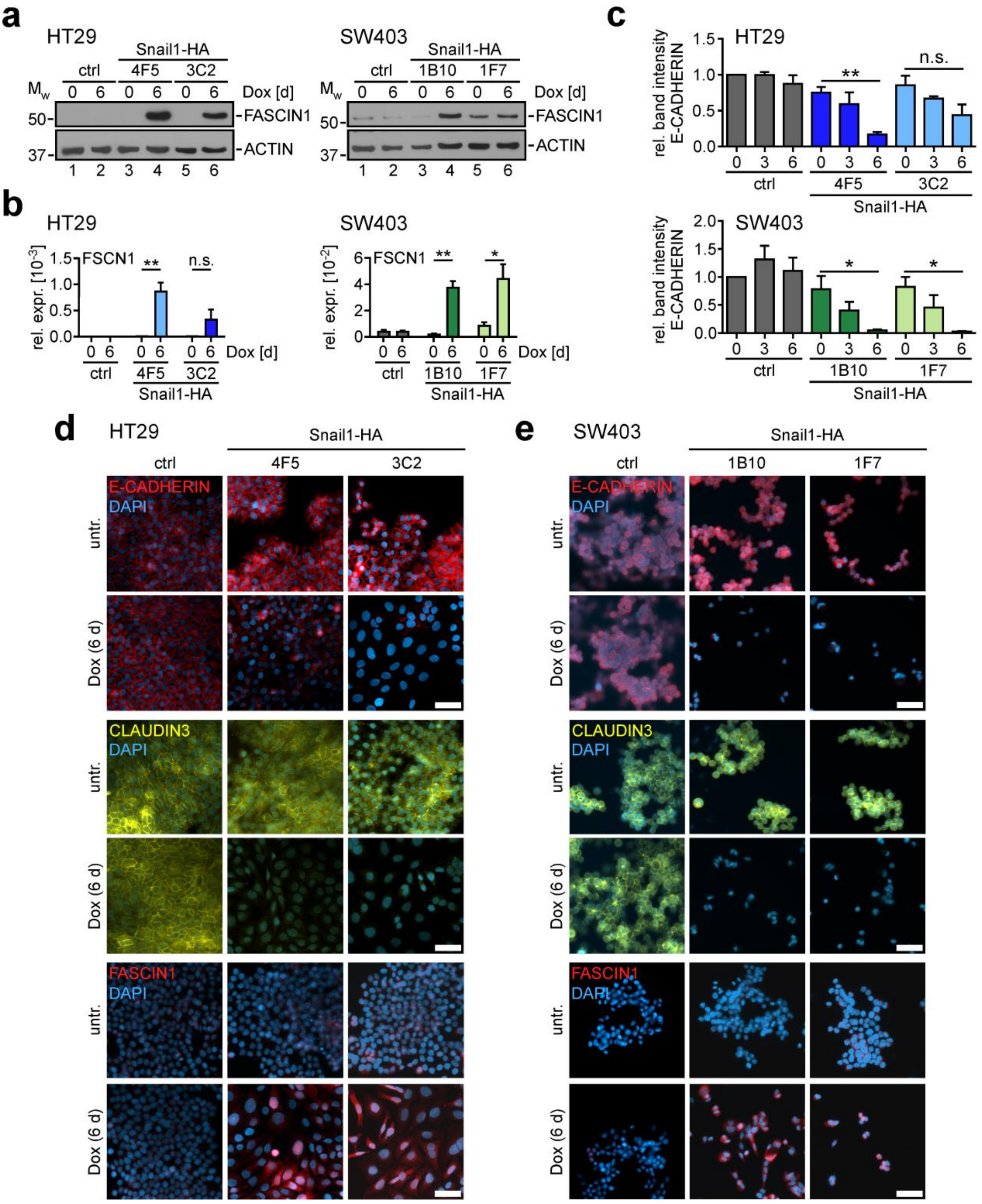

Supplementary Figure S5: Loss of epithelial markers and gain of the mesenchymal marker FASCIN1 in HT29 and SW403 *SMAD4*^{mut} CRC cells upon expression of Snail1-HA. (**a**) Analyses of protein expression by immunoblotting in control (ctrl) cells and Snail1-HA expressing cell clones derived from HT29 cells (left) and SW403 cells (right) treated with Dox as indicated. Positions of molecular weight (Mw) standards in kDa are indicated on the left. ACTIN served as loading control. (**b**) Analyses of FASCIN1 mRNA expression by qRT-PCR in control (ctrl) cells and Snail1-HA expressing cell clones derived from HT29 cells (left) and SW403 cells (right) treated with Dox as indicated. Relative gene expression (rel. expr.) was calculated by normalizing to the expression of *GAPDH*. Plotted is the mean + SEM; $n = 3$. Two-tailed student's t-test; *: p-value < 0.05, **: p-value < 0.01, n. s.: not significant. (**c**)

Quantification of E-CADHERIN levels in the immunoblot experiments related to Figures 2b and 2d. For each condition, densitometry values were determined and normalized to the intensity of the respective loading control bands. Values are shown relative to those measured in untreated control (ctrl) cells. Plotted is the mean + SEM; $n = 3$. Two-tailed student's t-test; *: p-value < 0.05, **: p-value < 0.01. (**d, e**) Immunofluorescence stainings of (d) HT29-ctrl cells and HT29-Snail1-HA clones 4F5 and 3C2, and (e) SW403-ctrl cells and SW403-Snail1-HA clones 1B10 and 1F7. Cells were treated with or without Dox for 6 days prior to staining with antibodies against E-CADHERIN, CLAUDIN3, and FASCIN1. DAPI was used to visualize nuclei. For each condition one representative image is shown. Scale bars: 50 µm. $n = 2$.

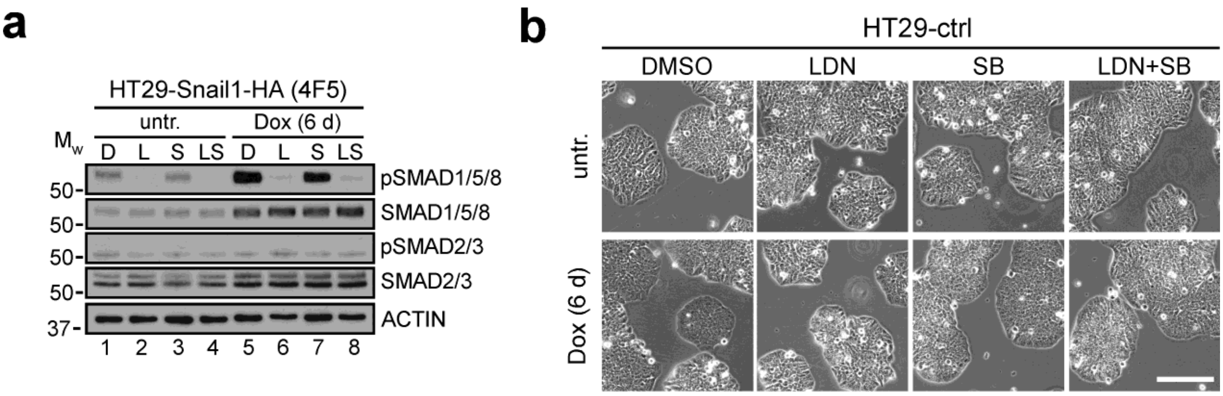

Supplementary Figure S6: Effects of BMP and TGFβ receptor inhibition on pSMAD levels in HT29-Snail1-HA cells. (**a**) Analyses of protein expression by immunoblotting in HT29-Snail1-HA clone 4F5 cells treated with Dox and DMSO (D), LDN193189 (L), SB431542 (S), or a combination of LDN and SB (LS) as indicated. Positions of molecular weight (M_w) standards in kDa are indicated on the left. One representative loading control of ACTIN is shown for reasons of simplicity. All loading controls corresponding to the depicted protein detections are given in Figure S9c. (**b**) Representative phase contrast images of the HT29-ctrl cells treated with Dox and DMSO, LDN193189 (LDN), SB431542 (SB), or a combination of LDN and SB as indicated. Scale bar: 200 µm.

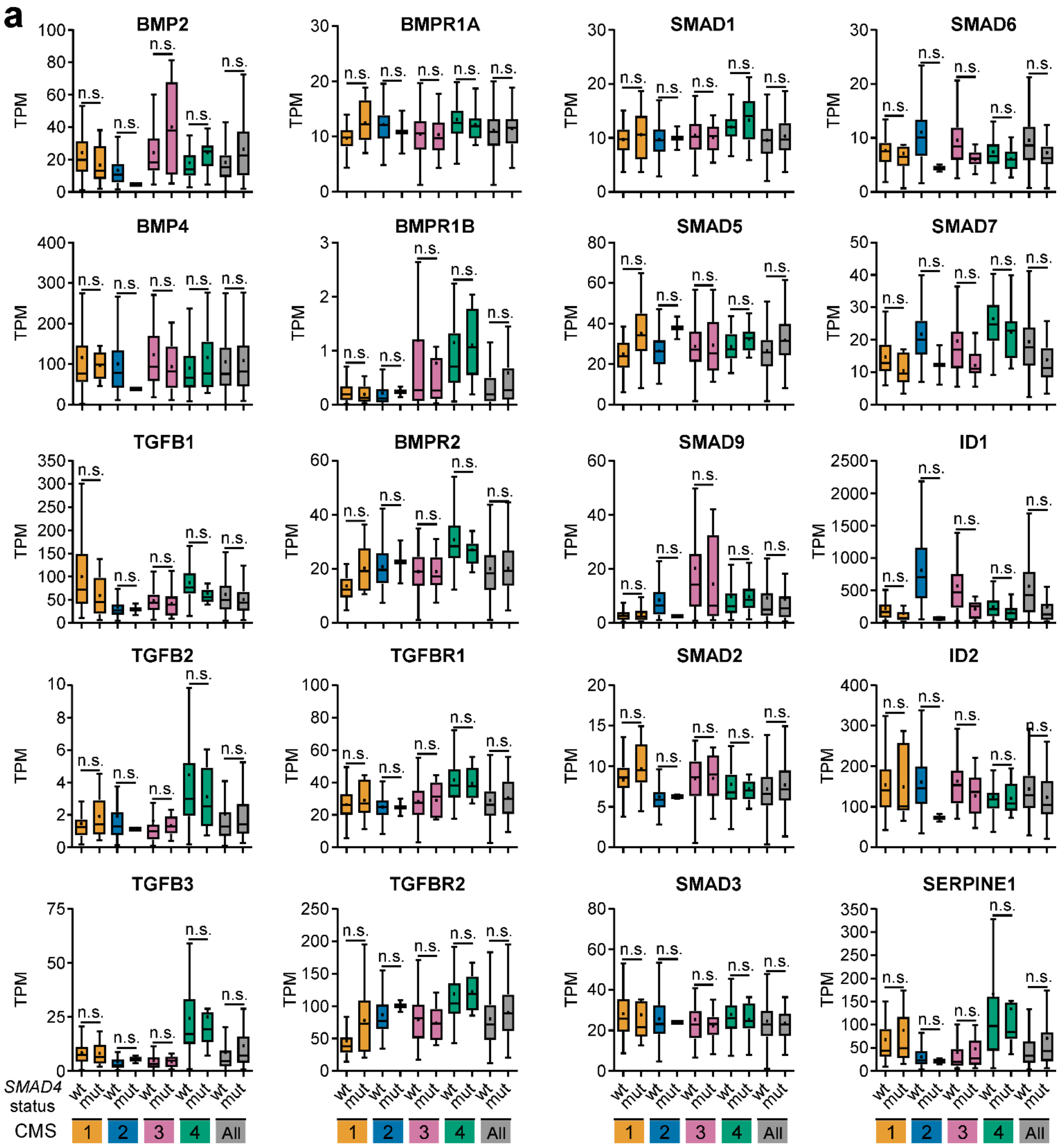

Supplementary Figure S7: Expression of TGFβ superfamily pathway components in *SMAD4*^{wt} and *SMAD4*^{mut} CRC patient samples. (**a**) Expression levels of TGFβ superfamily pathway components in colon adenocarcinoma (COAD) samples from The Cancer Genome Atlas (TCGA) database. Samples were grouped by their consensus molecular subtype (CMS) and further divided based on their *SMAD4* mutation status. Sample numbers for each condition are listed in Figure 6b. Dots in the box plots indicate average values. Whisker lengths are based on the Tukey method. Outliers are not plotted but were considered for calculating significance. Significance was determined by differential gene expression analysis with the *limma* package in R/Bioconductor; n.s.: not significant (adj. p-value ≥ 0.05). TPM: transcripts per million.

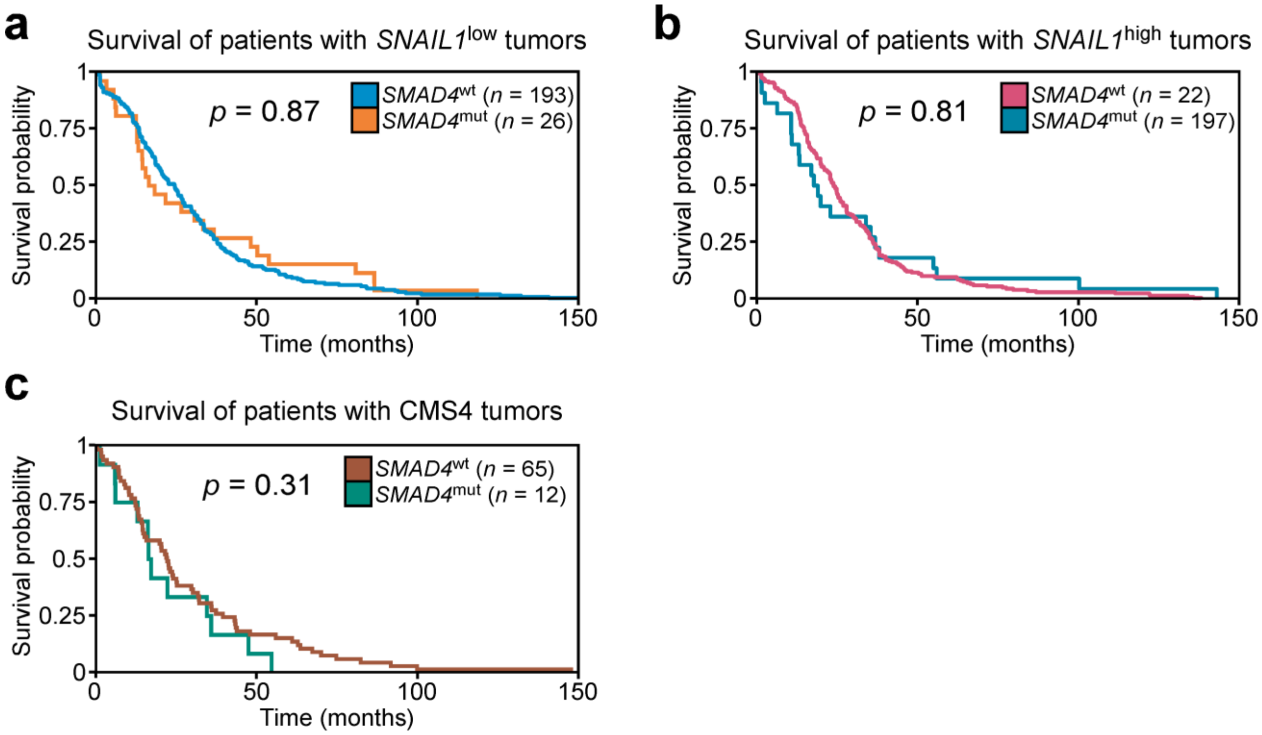

Supplementary Figure S8: (**a, b**) Kaplan-Meier curves showing the survival probability of CRC patients over time. Colon adenocarcinoma (COAD) patient samples from The Cancer Genome Atlas (TCGA) were first separated into two groups based on their expression of *SNAIL1* and then further stratified by their *SMAD4* status. Log-rank test was used to determine significance of differences in survival probability; *p*-values are indicated. (**c**) Kaplan-Meier curves showing the survival probability of CRC patients over time. Colon adenocarcinoma (COAD) patient samples from The Cancer Genome Atlas (TCGA) that were classified as consensus molecular subtype 4 (CMS4) were separated into two groups according to their *SMAD4* status. Log-rank test was used to determine significance of differences in survival probability; *p*-values are indicated.

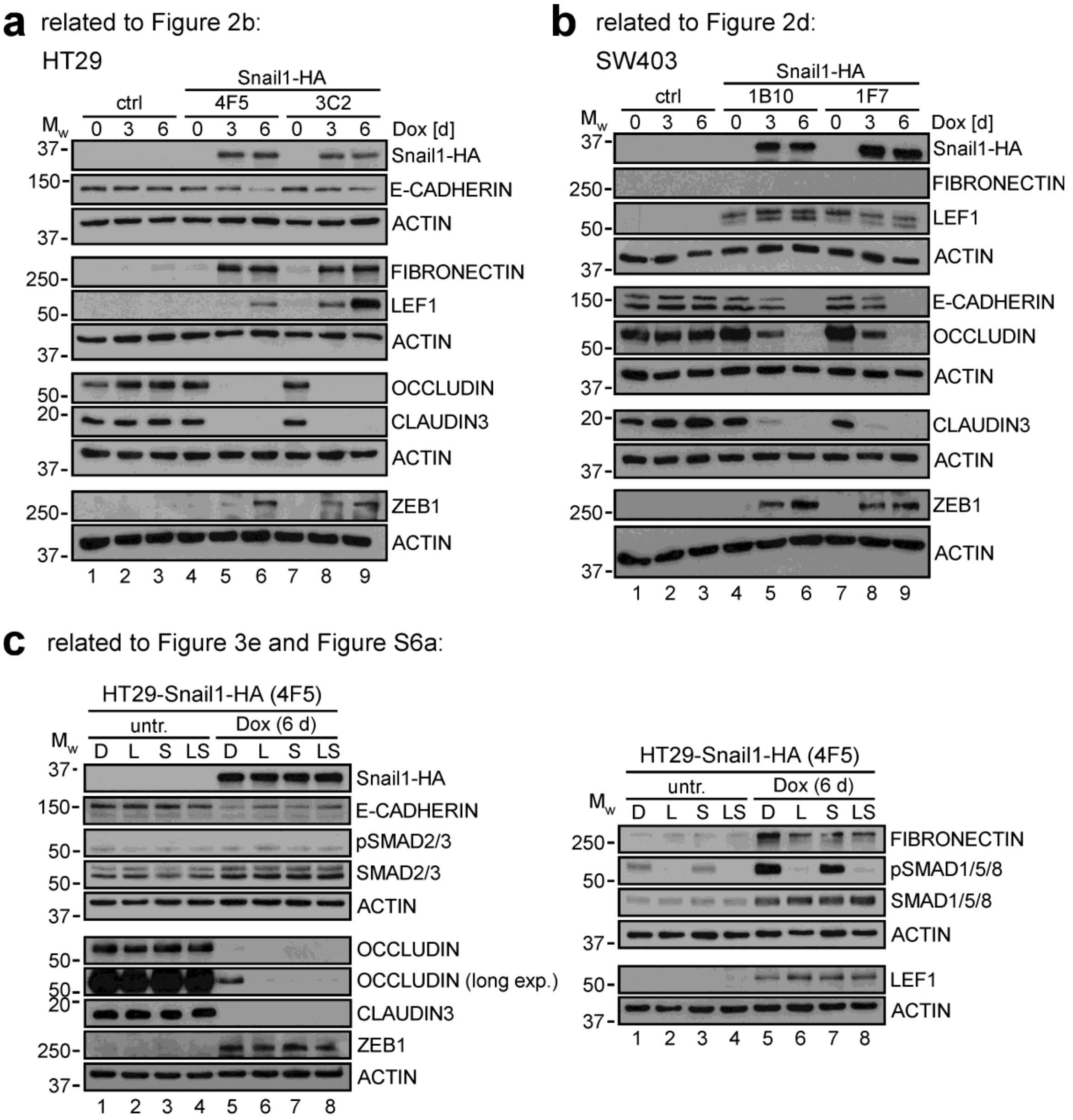

Supplementary Figure S9: Immunoblots with all corresponding loading controls. (**a-c**) Analyses of protein expression by immunoblotting in the cell lines denoted after treatment as indicated. ctrl: control; untr.: untreated; D: DMSO; L: LDN193189; S: SB431542; LS: LDN + SB. Positions of molecular weight (M_W) standards in kDa are indicated on the left. Plots are related to the indicated figures in the main manuscript. For each set of protein detections, the corresponding loading control of ACTIN is shown below.
